# Nurse‐Led Models of Service Delivery for Skin Cancer Detection: A Systematic Review

**DOI:** 10.1111/jan.16854

**Published:** 2025-04-01

**Authors:** Leila Kattach, Heidi Singleton, Steven Ersser, Debbie Holley, Ian Pearson, Abdulrahman Shadeed

**Affiliations:** ^1^ Department of Nursing Science, Faculty of Health & Social Science Bournemouth University Poole England; ^2^ University Hospital Dorset NHS Foundation Trust Poole England; ^3^ South West London and St George's Mental Health NHS Trust London England

**Keywords:** advanced practice, assessment, clinical effectiveness, clinical nurse specialist, dermatology, evidence‐based practice, health education, nurse practitioners, nursing diagnosis, policy

## Abstract

**Aim:**

To consolidate evidence on nurse‐led models for skin cancer detection by focusing on their roles, comparing their effectiveness to physician‐led care and highlighting any value‐added benefits.

**Design:**

Systematic review methodology with narrative synthesis.

**Data Sources:**

MEDLINE Complete, PubMed, Embase, CINAHL Complete, ScienceDirect, Scopus, BNI, LILACS, PsycINFO, Trip Medical Database, ERIC, EThOS, CDSR, WoS, Google Scholar, ClinicalTrials.gov, ICTRP, CENTRAL and the website ‘Getting It Right First Time’.

**Methods:**

This review adhered to the Preferred Reporting Items for Systematic Reviews and Meta‐Analyses checklist. Studies between January 1992 and September 2024 were evaluated using the Joanna Briggs Institute Critical Appraisal Checklists. The search encompassed both peer‐reviewed and grey literature; however, no grey literature met the inclusion criteria.

**Results:**

Of the 6680 records screened, six studies met the inclusion criteria, involving 3325 patients across England, New Zealand and the United States. These studies focused on nurse‐led models of care for skin cancer, assessing outcomes such as diagnostic accuracy, treatment effectiveness, cost savings, waiting times, access to care and patient satisfaction. While none directly compared nurse‐led to dermatologist‐led models, one study demonstrated comparable diagnostic accuracy between nurses and ophthalmologists. Nurse‐led models were shown to effectively substitute for or complement physician‐led care, though only one study was authored by a nurse consultant, highlighting a gap in nursing‐led research. Service users favoured community‐based, nurse‐led care for its accessibility, convenience and cost‐effectiveness, with health education noted as an added benefit in one study.

**Conclusion:**

Nurse‐led models demonstrate potential for high diagnostic accuracy in skin cancer, effective treatment delivery and enhanced patient education on skin self‐examination. While role delineation remains a challenge, nurses play a critical role in supporting dermatologists in addressing the increasing referral demands associated with skin cancer care.

**Trial Registration:** The systematic review protocol (registration number: CRD42023448950) was developed in collaboration with a patient representative with lived experience of melanoma, alongside academic experts in dermatology nursing and specialist; dermatology clinicians.

**Patient Contribution:**

A patient representative with lived experience of melanoma contributed to the review protocol.

**Policy and Practice Implications:**

- Training and Competency Development: Completing nationally recognised dermatology nursing qualifications beyond the Advanced Clinical Practice pathway and practical training to extend assessment, diagnostic and treatment skills are essential for autonomous practice in dermatology. Specific skills in nurse‐led skin cancer care are vital to ensure clinical competency.
- Dermatology Nurse Consultant Training Programme: Policies should prioritise nationally recognised Advanced Nurse Practitioner to Dermatology Nurse Consultant Training Programmes focusing on assessment, diagnostic and treatment skills. A structured, portfolio‐based approach to training is crucial for achieving competency and enabling autonomous practice in dermatology, supporting the delivery of high‐quality care.
- Support for Community‐Based Care: Policy‐level support for community‐based care is critical, particularly in rural or underserved regions. These models reduce patient travel, improve timely care access and provide training opportunities for rural clinicians, offering a viable alternative to hospital‐based services.
- Standardising Nurse‐Led Models: Developing national or international guidelines is essential for scaling nurse‐led models. Standardisation allows these models to adapt to the specific needs of local services while maintaining high standards of care.
- Delivering Comprehensive Care: Nurse‐led models show promise in delivering standard care comparable to physician‐led services for specific components of the skin cancer care pathway. They also provide value‐added care benefits, such as tailored patient education, enhancing outcomes and satisfaction.

**Impact Statement:**

Nurse‐led models demonstrate diagnostic accuracy in identifying skin lesions, including skin cancer, while contributing to treatment, patient education and follow‐up care. Despite their growing role in skin cancer management, greater dissemination and publication of their outcomes are needed to inform clinical practice. This review highlights the importance of standardising nurse‐led approaches into scalable frameworks to support dermatologists, enhance patient outcomes and ensure consistent care standards in skin cancer. Further evaluation is required to assess their efficiency, cost‐effectiveness and implementation across diverse healthcare settings.

**Summary**

- What is already known
  ○ The role of nurses specialising in skin cancer varies widely, indicating diverse nurse‐led models across different global contexts.
  ○ The scarcity of comprehensive analyses on the effectiveness, efficiency and patient outcomes associated with nurse‐led models for managing skin cancer.
- What this paper adds
  ○ Although no studies directly compared nurse‐led care to dermatologist‐led care, several studies demonstrated nurses' diagnostic accuracy in addition to their proficiency at identifying previously unsuspected lesions.
  ○ Various nurse‐led models have been identified as valuable in supporting physicians across different elements of the skin cancer care pathway.
  ○ Advocating for and standardising nurse‐led models in skin cancer care is crucial, enabling services to scale, adapt and tailor these models to support dermatologists in managing high referral demands, while maintaining a consistent standard of care internationally.
  ○ Rigorously designed studies are needed to further evaluate the resource efficiency, cost‐effectiveness and additional value‐added care provided by nurse‐led models.

## 1 Introduction

Skin cancer stems from cellular DNA damage that instigates mutations resulting in the dysregulated proliferation of aberrant skin cells within the epidermis (Hasan et al. 2023). These mutations propagate rapidly, ultimately forming malignant tumours known as skin cancer. The two principal categories of skin cancer are melanoma, originating from melanocytes and potentially fatal if diagnosed late and non‐melanoma skin cancers, including basal cell carcinoma, squamous cell carcinoma, and rarer variants (Berry 2016; Cancer Research UK 2022; 2023).

Individuals with lighter skin tones are predominantly affected, as their lower levels of photoprotective melanin make them more susceptible to ultraviolet radiation and, consequently, skin cancer (Garbe et al. 2021; Padovese et al. 2018; Schadendorf et al. 2018). Ultraviolet radiation, the most prevalent yet modifiable cause of skin cancer, has been well‐documented (D'Orazio et al. 2013; Schadendorf et al. 2018). Early detection improves the prognosis for non‐melanoma skin cancer (Didona et al. 2018), and thinner melanomas lead to better treatment outcomes (Swetter and Geller 2014). Skin cancer ranks as the 17th most common cancer globally and is the 14th most common cancer in both men and women (World Cancer Research Fund 2024). In 2022, 331,722 new cases of skin cancer were reported worldwide (World Cancer Research Fund 2024).

Cutaneous melanoma, a significant subset of skin cancer, accounts for approximately 287,723 cases annually, representing 1.6% of all newly diagnosed malignant cancers (Bray et al. 2018). Melanoma is also responsible for 60,712 deaths each year, contributing to 0.6% of global cancer‐related mortalities (Bray et al. 2018). Non‐melanoma skin cancer comprises 5.8% (1,042,056) of all new cancer cases annually and accounts for 0.7% (65,155) of cancer deaths (Bray et al. 2018). The global burden of skin cancer, with notably high incidence rates in countries such as Australia, the United States and Germany (World Cancer Research Fund 2024), highlights the urgent need for innovative care models adaptable to diverse healthcare systems worldwide.

Climate change has numerous environmental consequences, including rising global CO_2_ emissions, sea‐level rise, increasing temperatures and extreme weather events, all of which profoundly impact human health (Costello et al. 2009; Watts et al. 2021). Ozone layer depletion, driven by climate change, increases exposure to harmful ultraviolet radiation, particularly ultraviolet B radiation (Watson et al. 2024), which contributes to the rising incidence of skin cancer (Flynn et al. 2023a). Air pollutants, such as polycyclic aromatic hydrocarbons and phthalates, exacerbate this issue by inducing inflammation and disrupting skin barrier functions, further contributing to skin carcinogenesis (Flynn et al. 2023a). Flynn, Johnson, and Miller (2023b) reviewed the impact of climate change on cutaneous carcinogenesis, highlighting the rising incidence of skin cancer and its link to ultraviolet radiation. The study also outlined strategies for dermatologists to reduce carbon footprints and lead in climate advocacy.

Recognised by the World Health Organization (2023) as the most significant threat to human health, climate change is associated with deteriorating health outcomes, emphasising the critical need for comprehensive mitigation effort (Rocque et al. 2021). Although climate change's role in skin cancer is well‐recognised, further longitudinal studies are needed to quantify its specific impact across different regions and populations (Rocque et al. 2021). Meanwhile, emerging technologies, such as artificial intelligence, show promise in enhancing skin cancer detection and diagnosis, complementing both traditional and nurse‐led care (Stanford Medicine 2024). However, the adoption of artificial intelligence faces significant barriers, including a lack of robust clinical trials, challenges in workflow integration and insufficient evidence supporting consumer‐focused applications (Brancaccio et al. 2024).

Nurses play a recognised and pivotal role across health care sectors, being the ‘backbone’ of healthcare worldwide and delivering care in diverse roles and contexts (The Lancet 2019). Nurses and midwives constitute nearly 50% of the global health workforce and of the 43.5 million health workers globally, an estimated 20.7 million are nurses and midwives (World Health Organization 2017). Nursing is crucial for addressing demographic changes and growing healthcare demands (The Lancet 2019). Climate change necessitates a holistic approach to care, which nurses are well positioned to provide (The Lancet 2019). Nurse‐led clinics can effectively expand capacity for managing non‐communicable diseases (Doherty et al. 2018; The Lancet 2019) and offer effective, cost‐efficient care (Doherty et al. 2018). They are also adaptable and scalable, making them well‐suited to address the global demand for skin cancer services, particularly in low‐resource settings with limited physician availability.

Advanced practice nursing, an innovative and evolving field, aims to address care gaps and the increasing demand for healthcare services, driven by global challenges like climate change, an ageing population and rising non‐communicable diseases (Ladd et al. 2020). However, these roles have emerged unequally worldwide in response to local care needs, lacking consistent support for extended roles (Kilpatrick et al. 2023). Advanced nurse practitioner‐led care, which has increasingly replaced physician‐led care in various settings, is well received by patients (Htay and Whitehead 2021). Research on advanced nursing practice and its association with improved patient outcomes continues to grow, highlighting the significant contributions these nurses make across diverse healthcare settings (Poghosyan and Maier 2022). Nurse‐led initiatives can improve the uptake of early cancer detection, increase understanding, promote confidence in early detection and enable timely identification of precancerous lesions (Li et al. 2020). Studies show that patients widely accept nurse‐led case management (McParland et al. 2022).

Educating and engaging patients in managing their care has been shown to enhance patient‐centred outcomes, such as improved treatment adherence and quality of life (Doherty et al. 2018). Dermatology Clinical Nurse Specialists have been found to foster patient trust and engagement through the provision of tailored education, training and psychosocial support (van Os‐Medendorp et al. 2020). A dermatology nurse‐led education programme developed in the United Kingdom, involving group education and social learning for chronic dermatoses, was positively received by parents of affected children. Preliminary findings indicated positive clinical outcomes and improvements in quality of life (Ersser et al. 2013; Jackson et al. 2014). Nurse‐led care has been shown to have the potential to benefit not only service users but also healthcare services (Doherty et al. 2018; Htay and Whitehead 2021). Healthcare organisations globally implement varying nurse‐led care models, which reflect diverse patient needs, health system structures, clinician preferences (Loescher et al. 2011) and available resources.

This systematic review aims to address the growing demand for skin cancer services by collating, examining, appraising, and synthesising available evidence to assess whether nurse‐led models for skin cancer detection and management are as effective as traditional physician‐led care. Specifically, this review seeks to answer the following questions:

- Do nurse‐led models of service delivery for skin cancer provide one or more of the following components: assessment, treatment or education?
- Are the different nurse‐led models effective compared to physician‐led care (standard care)?
- Compared to standard care, do the models offer additional value‐added care for patients?

## 2 Methods

This systematic review was conducted in accordance with the Preferred Reporting Items for Systematic Reviews and Meta‐Analyses (PRISMA) 2020 guidelines (Page et al. 2021) for reporting systematic reviews and their abstracts. The completed PRISMA checklists specific to this study are available in Appendix S1. Furthermore, the review was registered with PROSPERO (registration number: CRD42023448950).

The protocol was developed in collaboration with a patient representative with lived experience of melanoma, academic experts and specialist dermatology clinicians.

### 2.1 Eligibility Criteria

Studies were selected based on the following criteria:

#### 2.1.1 Types of Studies

Except for pilot studies, all qualitative, quantitative and mixed‐methods studies were considered. The selection process was not restricted to peer‐reviewed publications, and the search included grey literature, ensuring a comprehensive inclusion of relevant information.

#### 2.1.2 Types of Participants

Service users of all age groups and all skin types according to the Fitzpatrick (1988) skin types I–VI, requiring skin cancer assessment, treatment and/or education.

#### 2.1.3 Types of Interventions/Comparators

The review focused on studies of nurse‐led models or interventions, comparing them to physician‐led care, as well as models delivered in tandem with or replacing physician‐led care. The included studies examined nurse‐led models that provided one or more of the following components: assessment, treatment or education for skin cancer. Various treatment modalities were included, such as photodynamic therapy, skin surgery, cryotherapy, topical treatments and educational interventions.

#### 2.1.4 Types of Outcomes

Nurse‐led models were evaluated across several outcomes based on the available data within the included studies. These outcomes included whether a full skin examination was performed and whether subsequent treatment was provided. The reliability of nurse‐led diagnostics was assessed by comparing the diagnostic accuracy of assessments conducted by nurses with those performed by physicians. The review also assessed the timeliness of care delivery, encompassing the period from assessment to diagnosis, initiation of treatment, referral, or discharge, as well as cost‐effectiveness. The review identified value‐added care for patients provided in addition to standard care equivalence and their impact on patient satisfaction and outcomes. Outcomes were collected as reported in individual studies and compared when similarities were observed.

#### 2.1.5 Types of Setting

Selected studies were not limited to hospital settings or restricted by medical specialty, ensuring inclusion of research across various medical fields. No restrictions were applied regarding the type of skin cancer, enabling a comprehensive review.

### 2.2 Information Sources

#### 2.2.1 Electronic Searches

A comprehensive literature search was undertaken to identify both published and unpublished studies across relevant electronic databases, which included: MEDLINE Complete, PubMed, Embase, CINAHL Complete, Science Direct, Scopus, British Nursing Index, Latin American and Caribbean Health Sciences Literature, PsycINFO, Education Resource Information Centre, Trip Medical, Electronic‐Theses Online Service, Web of Science and the Cochrane Database of Systematic Reviews. Trial registries were also searched, including ClinicalTrial.gov, the World Health Organisation International Clinical Trials Registry Platform and Cochrane Central Register of Controlled Trials. The search engine Google Scholar and the website ‘Getting It Right First Time’ (GIRFT 2021) were also searched.

#### 2.2.2 Searching Other Resources

Grey literature, such as abstracts and conference papers, was requested directly from international dermatology nursing organisations via email. To ensure comprehensive coverage, the citations and reference lists of selected studies were thoroughly examined to identify additional relevant papers (Bowen 2008).

### 2.3 Search Strategy

The search strategy applied the PICO framework, comprising: Population, Intervention, Comparator/s, Outcomes (Higgins and Green 2011) and the search terms included a combination of the keywords as well as their synonyms. The key search terms were:Skin cancer AND Nurs* or Nurs* led.

Medical Subject Headings indexing, Boolean operators, wildcards and truncation were systematically applied where available (see Appendix [Link], [Link] for details on searches and search dates).

### 2.4 Data Collection and Analysis

#### 2.4.1 Selection of Studies

All retrieved abstracts, studies and citations were collated in the EndNote reference management database. Duplicates were systematically removed, and a staged review process began with a preliminary screening of titles and abstracts in EndNote to exclude irrelevant articles (e.g., studies focusing on breast cancer). Any articles that mentioned skin cancer or nurse‐led care in the title or abstract were transferred into the software Covidence. Covidence was used to streamline the review process and adhere to best practices for systematic review methodology. The specific inclusion and exclusion criteria, along with detailed definitions of eligibility parameters, are outlined in Table 1. Using Covidence, two reviewers (1 and 6) independently reviewed the titles and abstracts of each paper, and irrelevant papers were removed. The full texts of the remaining articles were independently reviewed, with selection justifications recorded. Any discrepancies regarding eligibility were resolved through group discussion (1–4), and consensus was reached.

**TABLE 1:** Eligibility criteria applied through covidence screening.

Eligibility criteria			
Category	Include	Exclude	Exclusion reason
Population	Patients with skin lesions requiring assessment. Patients with a history of skin cancer requiring follow‐up. Immunocompromised, high‐risk patients requiring full‐body skin examinations for potential skin cancer. Patients referred for primary prevention education (e.g., sun protection and awareness) or secondary prevention (e.g., early detection and treatment). Human subjects of all ages referred by general practitioners or equivalent providers	Patients without skin lesions. Education programs targeted at health professionals, nursing students, or nurses instead of patients. Studies focusing on nurse‐led models of service delivery related to immunotherapy or targeted therapy for metastatic melanoma, specifically examining potential adverse events, rather than skin cancer detection. Studies evaluating combined clinician groups without separate analysis of nurses. Studies unrelated to skin cancer or not involving nurse‐led model.	Descriptive paper only. Study type does not match eligibility criteria—pilot study. Audit or service evaluation. Nurses were involved in the study but describing the structure or evaluating the effectiveness of a nurse‐led service delivery model or intervention was not the purpose of the study. Unable to obtain full text. No nurses were involved in the study. No nurse‐led service delivery model or nurse‐led intervention was included. The effectiveness and/or efficiency of the nurse led model of service delivery or nurse‐led intervention was not assessed. The nurse‐led model of service delivery is related to immunotherapy or targeted therapy for metastatic melanoma examining potential adverse events and not skin cancer detection. The study evaluates an intervention, educational session, course, or multi‐form training delivered to health professionals or students rather than patients or individuals with skin lesions. The study is generic and not specific to skin cancer, e.g., all types of surgery (not just skin cancer removal) or all types of cancer (e.g., breast, colorectal, etc.). The study was an assessment of nurse's competence and not a nurse‐led model of service delivery or a nurse‐led intervention. Wrong participant group e.g. not patients or people requiring skin lesion assessment. Wrong intervention. Wrong outcomes.
Intervention/exposure	Studies focused on nurse‐led service delivery models for skin cancer detection, including assessment, treatment, or education by registered nurses. Studies specifically related to a nurse‐led service delivery model for skin cancer detection that provides assessment, treatment and/or education delivered across all health care settings and internationally.	Nurse‐led service delivery model not specific to ‘skin cancer’ e.g., related to general cancer care or colorectal cancer, breast cancer and skin cancer combined. Service delivery model for skin cancer detection that provides assessment, treatment and/or education that is delivered by only non‐nursing clinicians with no reference to nurse‐led service e.g., pharmacist‐led vs. physician led care. Nurses cannot be grouped with other allied health professionals.	
Comparator/context	Physician‐led care, standard practice, or gold standard care.	Comparisons other than physician‐led care, standard practice, or gold standard care (e.g., comparisons to physician associates without separate comparison to physicians).	
Study characteristics	Studies defined as: ○ Randomised controlled trials. ○ Cluster randomised controlled trials (by nurses and physicians). ○ Quasi‐experimental and cross over studies. Relevant qualitative studies as they may offer greater understanding related to the psychological/behavioural dimensions of the review i.e., effectiveness, behaviour change and/or acceptability and examples include: ○ Descriptive. ○ Observational. ○ Correlational. ○ Population‐based. ○ Case study. ○ Survey. ○ Other relevant study type.	Not applicable.	
Other	Not applicable.	Studies unavailable in full text. Studies with missing data preventing review (authors will be contacted before exclusion). Abstracts or conference proceedings lacking sufficient study details.	

#### 2.4.2 Inclusion Criteria

The literature search focused on full‐text studies in English, made available between 1 January 1992 and 10 September 2024. The year 1992 was selected as the starting point because it marks a significant milestone in the advancement of nursing practice and expanded roles (Rushforth and McDonald 2004). The search included peer‐reviewed and grey literature to ensure comprehensive coverage and reduce publication bias.

#### 2.4.3 Exclusion Criteria

Excluded studies included those of nurse‐led models delivering care to cancer patients with different types of cancers combined (e.g., skin and lung cancer patients consulted in one clinic) and models administering immunotherapy or targeted therapy to patients with metastatic melanoma, (e.g., managing side effects or adverse events). Pilot studies were excluded, and studies of models delivered by non‐medical registered professionals (e.g., pharmacists or physician associates) or unregistered professionals (e.g., health care assistants), and studies with outcome data from combined groups of registered or unregistered non‐medical professionals.

#### 2.4.4 Assessment of Risk of Bias in Included Studies

Grey literature was included in the search strategy; however, no sources met the inclusion criteria and were consequently not evaluated. If relevant grey literature had been identified, it would have been appraised for quality using the Authority, Accuracy, Coverage, Objectivity, Date and Significance checklist (Tyndall 2010). The papers were meticulously read and critically appraised (Aveyard and Bradbury‐Jones 2019) to assess the methodological robustness, reliability, focus and overall quality of the studies in relation to the research questions. A thorough evaluation was conducted to identify and address potential biases in study design, conduct, analysis and presentation (Moola et al. 2020). Two authors (1 and 2) independently evaluated the methodological rigour and quality of the included studies using the Joanna Briggs Institute Critical Appraisal Tools for Case Series (Munn et al. 2019), Cohort (Moola et al. 2020), Case Control (Moola et al. 2020) and Quasi‐Experimental studies (Tufanaru et al. 2020). The details of the quality appraisal are provided in Appendix S3.

The overall quality grading and justification for each study are detailed in Table 2, while Table 3 summarises the strengths and weaknesses of each study.

**TABLE 2:** Quality appraisal of the included studies.

Articles number	Article 1	Article 2	Article 3	Article 4	Articles 5	Article 6
Author and title	Jones, C. and Mullen, L. A service evaluation of a nurse consultant led basal cell carcinoma clinic.	Mohite, A. A., Johnson, A., Rathore, D. S., Bhandari, K., Crossman, R., Mehta, P. and Ahluwalia, H. S. Accuracy of clinical diagnosis of benign eyelid lesions: Is a dedicated nurse‐led service safe and effective?	Clayton, T. H., Tait, J., Whitehurst, C., and Yates, V. M. Photodynamic therapy for superficial basal cell carcinoma and Bowen's disease.	Jones, L., Jameson, M., and Oakley, A. Remote Skin Cancer Diagnosis: Adding Images to Electronic Referrals Is More Efficient Than Waitlisting for a Nurse‐Led Imaging Clinic.	Lim. D., Oakley., A. M. and Rademaker, M. Better, sooner, more convenient: a successful teledermoscopy service.	Oliveria, S. A., Dusza, S. W., Phelan, D. L., Ostroff, J. S., Berwick, M. and Halpern, A. C. Patient adherence to skin self‐examination. effect of nurse intervention with photographs.
Overall quality, assessed using the appropriate JBI quality appraisal tool, Case Series (Munn et al. 2019) Cohort (Moola et al. 2020), Case Control (Moola et al. 2020) or Quasi‐Experimental (Tufanaru et al. 2020)	High	High	Moderate	Moderate	Low	Moderate
Justification for Overall Quality Grading	The inclusion criteria were clear and reliable and valid measurement methods were applied. Clinical information and outcomes were reported coherently despite insufficient reporting of demographics and aspects of statistical analysis.	The inclusion criteria are clear and reliable and valid measurement methods were used. The reporting of demographics and clinical information were comprehensive, and an appropriate use of statistical analyses was applied to the findings.	The study demonstrated a clear distinction between cause and effect, similarity in participant comparisons, and adequate follow‐up description. It lacked multiple pre‐and post‐intervention outcome measurements, clarity in treatment/care comparability, reliability in outcome measurement, and transparency in statistical analysis.	The two groups were recruited from the same population, but this process had the potential for selection bias as group allocation was determined by the general practitioner triaging referrals. Confounding factors were identified, and valid and reliable outcome measurements were used. Participants were free of the outcome at the start and sufficient follow‐up time allowed. However, clarity was lacking regarding exposure measurement and assignment, strategies to address confounding factors and whether the exposure was measured in a valid and reliable way.	There were differences between the groups recruited from the same population. It was not clear whether exposure was measured in a valid and reliable way. It was unclear whether confounding factors had been identified. Follow‐up time sufficiency, completeness, the reasons for loss to follow‐up, strategies to address incomplete follow‐up, and statistical analysis clarity were unclear despite valid and reliable measurement of exposure and outcome.	While this study demonstrated suitable matching and identification of cases and controls, and standard and valid outcome assessments, it lacked clarity in exposure measurement and confounding factors. The exposure period was insufficient to determine the long‐term benefit of the photobook on self‐skin examination for monitoring.

**TABLE 3:** Summary of the characteristics of the included studies.

Articles number	Article 1	Article 2	Article 3	Article 4	Articles 5	Article 6
Author and Title	Jones, C. and Mullen, L. A service evaluation of a nurse consultant led basal cell carcinoma clinic.	Mohite, A. A., Johnson, A., Rathore, D. S., Bhandari, K., Crossman, R., Mehta, P. and Ahluwalia, H. S. Accuracy of clinical diagnosis of benign eyelid lesions: Is a dedicated nurse‐led service safe and effective?	Clayton, T. H., Tait, J., Whitehurst, C., and Yates, V. M. Photodynamic therapy for superficial basal cell carcinoma and Bowen's disease.	Jones, L., Jameson, M., and Oakley, A. Remote Skin Cancer Diagnosis: Adding Images to Electronic Referrals Is More Efficient Than Waitlisting for a Nurse‐Led Imaging Clinic.	Lim. D., Oakley., A. M. and Rademaker, M. Better, sooner, more convenient: a successful teledermoscopy service.	Oliveria, S. A., Dusza, S. W., Phelan, D. L., Ostroff, J. S., Berwick, M. and Halpern, A. C. Patient adherence to skin self‐examination. effect of nurse intervention with photographs.
Year of publication	2014	2016	2006	2021	2012	2004
Designation of first author	Nurse consultant for skin cancer	Consultant ophthalmologist	Consultant dermatologist	Dermatology registrar	Specialist dermatologist and Mohs surgeon	Epidemiologist
Country and geographical area	Liverpool, England	Coventry, England	Lancaster, England	Waikato, New Zealand	Waikato, New Zealand	New York, United States of America
Study setting	Secondary care dermatology department.	Secondary care ophthalmology department.	Secondary care dermatology department or health centre in primary care.	Primary care for the initial consultation. Followed by: Option 1: a general practitioner‐initiated referral to a community nurse‐led virtual lesion clinic for imaging and then virtual secondary care input (dermatologist reviewing the images and patient history). Option 2: a general practitioner initiated direct referral to a suspected skin cancer pathway whereby images and a patient history was sent directly a dermatologist to assess.	Primary care with secondary care virtual assessment by a dermatologist and either referral to a community nurse‐led virtual lesion clinic or direct for a face‐to‐face appointment in secondary care.	Privately funded tertiary cancer centre.
Research method	Case Series	Case Series	Quasi‐experimental	Cohort	Cohort	Case–control
Aims of Study	To evaluate a new nurse consultant‐led basal cell carcinoma service within a dermatology outpatients department based in secondary care.	Primary objective: to evaluate the diagnostic accuracy and outcomes of an independent nurse‐led oculoplastic service that manages benign eyelid lesion referrals. Secondary objective: to investigate the correlation between the clinical and histological diagnosis of each benign eyelid lesion among different grades of clinicians (including nurses).	Primary objectives: (1) To identify if photodynamic therapy can be carried out successfully in the community for the treatment of superficial basal cell carcinoma and Bowens disease. (2) To compare the quality of care and convenience to patients of the two treatment strategies: 5‐aminolaevulinic acid photodynamic therapy performed in a community setting versus being administered in a hospital setting. Secondary objectives: Comparison of cost effectiveness of both treatment strategies Tolerability and side effects of the treatment.	To assess the efficacy and efficiency of direct teledermoscopy via general practitioner electronic referral to the suspected skin cancer pathway in comparison to a nurse‐led virtual lesion clinic pathway. Primary objective: to compare the time from receipt of the referral to return of the dermatologist advice. Secondary objectives: (1) to determine the diagnostic concordance between general practitioner and dermatologist (and histology if available) between the suspected skin cancer 2020 cohort and virtual lesion clinic cohorts from 2020 and 2016. (2) to identify (if any) incidental skin cancers found during follow‐up specialist appointments.	To review the efficiency and patient acceptance of a new community‐based teledermoscopy service by comparing the service to hospital‐based face to face skin lesion clinics.	To assess the impact of a brief, nurse‐delivered, randomised‐design intervention utilising digital photographs on patients' adherence to performing skin self‐examination.
Timeframe of study	12 weeks	6 years (2007–2012)	Reviewed at 8 weeks and 6 months post treatment	Virtual lesion clinic referrals from July to December 2016 and 2020 Suspected skin cancer referrals from July to December 2020	8 months	4 months
Participant (population)	118 patients (2 did not attend)	596 lesions on 471 patients	50 patients	2296 patients with 2934 lesions	300 patients (8 patients (7%) in the face‐to‐face group and 15 patients (7%) in the virtual lesion clinic group failed to attend)	100 participants (5 declined)
Participant selection	General practitioner referrals to dermatology in secondary care for patients with a skin lesion suspected to be a basal cell carcinoma. Adults.	All patients with eyelid lesions that were operated on within an ophthalmology service in secondary care. Adults.	Patients referred by a dermatologist for photodynamic therapy. Adults.	Referrals were identified by searching departmental records. For the 2020 suspected skin cancer and virtual lesion clinic cohorts, the keywords ‘lesion’ and ‘skin cancer’ were used and for the 2016 virtual lesion clinic cohort a unique coding identifier was used. Adults and children.	Patients referred to the dermatology service by their general practitioner with one or more skin lesions were triaged by the duty dermatologist and sent to either a traditional face to face clinic or a nurse‐led virtual lesion clinic. Adults and children.	Patients who attended the outpatient pigmented lesion clinic within a dermatology service at a private tertiary cancer centre. Adults.
Inclusion criteria	General practitioner referrals of patients with a lesion/s suspected to be a basal cell carcinoma needing assessment in secondary care.	Patients who underwent excision of suspected benign eyelid lesion/s.	Patients in good general health with a diagnosis of either Bowens disease or a superficial basal cell carcinoma with diagnosis confirmed via skin biopsy. A maximum of 5 lesions were treated per patient.	Lesions suspected of being skin cancer were referred by general practitioners for assessment.	A general practitioner requested a diagnosis and/or management plan for one or more skin lesion(s). One to six lesions, clearly identified by location and that were not in the hair‐bearing areas which included the scalp or on the genitalia.	People with five or more clinical dysplastic/atypical naevi (as determined by a physician), who were willing to have digital whole‐body photography and agreed to be randomised to an intervention arm.
Exclusion criteria	None mentioned	Lesions classified as chalazia, dermoids, caruncular and conjunctival lesions	Patients who are systemically unwell or have a recurrent lesion	Referrals for reasons other than a suspected skin cancer	None mentioned	Patients who were visually or physically impaired or had been previously photographed or given a photo book.
Method of recruitment of participants (e.g., phone, email, clinic patients, review of patient records, voluntary, paid, other)	Secondary care outpatients' National Health Service clinic—voluntary participation.	A retrospective analysis of electronic National Health Service patient records.	Secondary care dermatology clinic—voluntarily participation. The first 25 consecutive patients were added to the secondary care group and the second 25 consecutive patients were allocated to the primary care group.	A retrospective review of electronic referrals/patient records	Primary care/general practitioner practice. Patients referred to dermatology by their general practitioner with one or more skin lesions—voluntary participation	Patients were recruited from an outpatient dermatology pigmented lesion clinic at a private cancer centre—voluntary participation. Stratification by personal history of skin cancer (yes versus no) during enrolment and the participants were randomised to a group.
Data collection methods	A proforma was designed and included quantitative evaluation criteria. Data was collected through full skin examinations conducted by the nurse consultant, with any additional lesions identified being assessed, treated and appropriately coded. If surgery was performed, data were collected on the type of surgery and clinician who performed the surgery. Follow‐up data and other outcome data were collected. Qualitative patient testimonies were collected by the clinical nurse specialist telephone follow‐up clinics.	An analysis of electronic patient records of all patients who underwent excision of suspected benign eyelid lesions over a period of 6 years (2007–2012). All excised lesions were sent for histological confirmation. The pre‐excision clinical diagnosis of each lesion/s was recorded and then compared to the histological diagnosis.	Following the initial treatment all patients were asked to complete a questionnaire which included questions regarding the distance travelled to the treatment centre, mode, cost of transport and patient satisfaction. All patients were reviewed 8 weeks and 6 months after treatment by the dermatologist at their follow up appointments.	A retrospective review of referrals was conducted by searching departmental records using keywords/identifiers. Data was collected from the 2020 suspected skin cancer pathway referrals and 2016 and 2020 virtual lesion clinic referrals which included the number of referrals, the time from referral to advice, the number of lesions per referral and patient demographics. For the suspected skin cancer pathway cohort, image quality was recorded.	Participants were surveyed four weeks after their clinic appointment regarding satisfaction, waiting times, convenience and confidence in the virtual lesion clinic process. The surveys were posted to patients in both groups and a different survey was also sent to the referring general practitioners. A financial analysis was performed to determine the technique's economic viability. The total number of patient interactions for both groups were analysed, including visits required to make a diagnosis and to subsequent nurse treatment clinics.	A physician examination was conducted at baseline to collect information on the number of moles and dysplastic naevi. All patients then underwent whole‐body photography, and the nurse education module was also delivered. Self‐administered questionnaires containing items regarding demographics, skin self‐examination practices, skin cancer knowledge and awareness, and personal and family history of cancer were provided and completed by participants. Outcome assessment and evaluation occurred at three time points: (1) baseline; (2) post session, immediately after the delivery of the interventions; and (3) follow‐up at 4 months post‐baseline visit.
Ethical considerations as described by the authors	A letter of approval for the basal cell carcinoma service evaluation was granted by the Hospital Trust Research Ethics Clinical Audit department.	The study complied with the policies of the local institutional review board and adhered to the tenets of the Declarations of Helsinki.	The local ethics committee approved the study.	The Health and Disabilities Committee of New Zealand determined the study to be a service review and out of the scope for formal ethics approval.	This study was solely funded by the District Health Board. No other ethical considerations were mentioned.	Patients provided informed consent. No other ethical considerations were mentioned.
Clinic type and designation of nurses delivering the nurse‐led service.	Skin cancer nurse consultant–led basal cell carcinoma clinic.	Oculoplastic clinical nurse specialist‐led clinic.	Dermatology clinical nurse specialist‐led photodynamic therapy treatment clinic.	Nurse specialist/melanographer‐led community teledermoscopy virtual lesion clinic.	Nurse specialist/melanographer‐led community teledermoscopy virtual lesion clinic.	Dermatology trained registered nurse‐led teaching/educational intervention.
The findings	A full skin examination: performed by the nurse consultant. Additional unsuspected lesions were found in 29.1% (*n* = 34) of patients. 15.4% (*n* = 18) had further basal cell carcinomas, 1.18% (*n* = 1) malignant melanoma, and 1.18% (*n* = 1) squamous cell carcinoma. Including primary lesions and incidental findings, 3.53% (*n* = 3) patients were diagnosed with invasive malignant melanoma and 3.53% (*n* = 3) squamous cell carcinomas. 1.7% (*n* = 2) had incidental severe psoriasis and were referred for further care. Treatment: initiated by the nurse consultant and included cryotherapy, topical treatment (Aldara/Efudix), surgery and ‘no treatment’. The nurse consultant also performed surgery. Health education: provided by the nurse consultant but not detailed. Care equivalence to physician: not examined. Nurse‐led care delivered: care that would have otherwise been delivered by a consultant physician. Nurse consultant‐led care was found to deliver comprehensive care to 89% (*n* = 105) of patients with no input from a dermatologist. Cost effectiveness: care delivered was equivalent to a medical consultant and yet the funding required was less. Service user feedback: qualitative patient testimonies collected via telephone and follow‐up clinics, revealing positive feedback about the care received from the nurse consultant. Some patients not fully understanding the nurse consultant role. Value added care for patients: increased access and shortened waiting times.	A full skin examination: not mentioned. Only referred lesions were triaged by the nurse specialist. Treatment: performed by surgical removal of the lesion/s. Health education: not mentioned. Care equivalence to physician: patient demographics were comparable showing no statistical significance (*p* > 0.05) between physician and nurse. For all lesion subtypes combined, there was no statistical significance between the nurse‐led and the physician‐led service with the former achieving an overall diagnostic accuracy of 80.4% compared to 79.6% for the latter (*p* > 0.05). The missed malignancy rate was also comparable at 1.5% for the doctors and 1.1% for the nurses (*p* > 0.05). Cost effectiveness: the band 7 specialist nurse cost approximately 55% less per minor operations list than the physician which was an 18%–23% cost saving per theatre list of 8 patients. Service user feedback: not collected. Value added care for service users: not mentioned.	A full skin examination: not mentioned. Treatment: photodynamic therapy was delivered by the nurse. Health education: not mentioned. Care equivalence to physician: not examined. Nurse‐led care delivered: all 25 patients treated in local health centres were able to remain at home during the 5‐aminolaevulinic acid (topical treatment) absorption phase which resulted in a shorter treatment time at their local health centre. 100% (*n* = 25) of community treated patients were treated within 1–2 h and 88% (*n* = 22) within one hour. For patients treated in hospital, the treatment time was longer with 8% (*n* = 2) of patient's treatment time taking over 6 h to administer, 64% (*n* = 16) taking 3–6 h and 28% (*n* = 7) taking under 3 h. Photodynamic therapy was well tolerated with similar outcomes and cure rates in both groups. There was no difference between the outcomes of the two groups when assessed (to check for resolution) at 6 month follow up by the dermatologists. Cost effectiveness: the overall costs of the PDT treatment were lower in the group treated in primary care. In most cases this reflected the increase transport cost of attending hospital. Service user feedback: the authors asked each patient to complete a questionnaire revealing that patients expressed a preference for community‐based treatment over hospital settings, with many citing the inconvenience of having to cancel an entire‐day of engagements and needing transportation support for secondary care treatment. Value added care for patients: (1) patients preferred treatment administered in the community as the care was closer to home and convenient. (2) No requirement for a relative to attend hospital with patients. (3) Same standard of care with less cost to patient or their family.	A full skin examination: not mentioned. The nurses identified 107 incidental lesions in patients attending imaging clinics which implies that skin examination assessments were conducted. Treatment: delivered by the nurse was unclear but cryotherapy and topicals was mentioned. Patients for surgery were referred to a physician in the hospital. Health education: not mentioned. Care equivalence to physician: the authors concluded that the study met their primary objective to demonstrate a statistically significant reduction in time to dermatologist advice using the suspected skin cancer pathway in 2020 compared to the virtual lesion clinic in 2016 and 2020. The median time from referral to advice was 4 days compared to 42 days and 50 days, respectively (*p* < 0.001). There was a reduction in time to definitive treatment for the suspected skin cancer pathway compared to the 2016 and 2020 virtual lesion clinic. Both for time from referral assessment (or triage for patients referred to the virtual lesion clinic) and for the time from dermatologist advice to treatment. Dermatologists recommended ‘no further action’ for fewer lesions (56% *n* = 298) in the suspected skin cancer pathway cohort and 57% (*n* = 157) and 69% (*n* = 471) in the 2020 and 2016 virtual lesion clinic cohort respectively. The virtual lesion clinic nurse specialists delivered uniformly high‐quality images taken with standardised cameras. In the suspected skin cancer pathway group, 5% (*n* = 71) of lesions could not be diagnosed due to poor image quality. Cost effectiveness: not mentioned. Service user feedback: not collected. Value added care for patients: was not mentioned	A full skin examination: not mentioned. Treatment: administered by the nurses was topical treatment and cryotherapy. Health education: not mentioned. Care equivalence to physician: the mean waiting time for a face‐to‐face appointment was 114 days, compared to 39 days for the virtual lesion clinic, representing a 66% reduction in the mean waiting time (*n* = 75 days). At the start of the study, the mean waiting time for the face‐to‐face group was 66 days, compared to 37 days in the virtual lesion clinic group, a difference of 44% (*n* = 29 days). Despite being allocated fewer patients, the face‐to‐face clinic was unable to keep up with demand, peaking at a mean waiting time of 138 days. The mean month‐by‐month waiting times for the virtual lesion clinic remained stable, ranging from 30 to 48 days. Dermatologists continued to diagnose lesions in the virtual lesion clinic group while attending conferences overseas. A total of 210 lesions were assessed in the face‐to‐face group and 383 lesions in the virtual lesion clinic group. Most patients in both groups had a single lesion. Fewer patients seen at the virtual lesion clinic (36.5%) were recommended for treatment compared to those seen at the face‐to‐face clinic (60%). Additionally, 59% (*n* = 118) of the 200 patients referred to the virtual lesion clinic were discharged. Cost effectiveness: was demonstrated through a financial analysis, revealing a per‐patient cost saving of $42.00 in 2012 (approximately £21.50), equating to a 14% reduction in overall costs in the virtual lesion clinic. Costs were provided by the District Health Boards who paid a per‐patient contracted fee to the virtual lesion clinic provider. Value added care for patients: was evident from survey responses, which showed a preference for the virtual lesion clinic over face‐to‐face consultations, with patients rating their satisfaction on a scale from 1 (poor) to 5 (excellent). Value added care for service users: (1) convenience of care and care closer to home. The authors highlighted ‘Better, Sooner, “More Convenient” care’	A full skin examination: not performed by the nurses. Treatments: no treatments were administered by the nurses. A nurse‐led intervention was delivered with and without a photobook. Health education: the nurse‐led intervention provided educational materials for patients and delivered education which included: (1) The cutaneous characteristics of melanomas. (2) Risk factors of melanoma. (3) Methods for conducting self‐skin examination with equipment or a partner to assist. Care equivalence to physician: not examined. Nurse‐led care delivered: Group A (teaching intervention with a photobook) and Group B (teaching intervention without a photobook), 86% (*n* = 42) and 84% (*n* = 43) respectively, completed the 4‐month follow‐up. To assess skin self‐examination, a standardised question was used: ‘How many times in the past 4 months did you (or someone else) thoroughly examine your skin? By thorough we mean looking at all the different areas of your skin deliberately and systematically’. In Group A, 10.2% (*n* = 5) of the patients at baseline reported self‐skin examination three or more times during the past 4 months, while 61.2% (*n* = 30) reported self‐skin examination three or more times at the 4‐month follow‐up (*p* 0.039 for paired comparison). In Group B almost 20% (*n* = 10) of the patients at baseline reported self‐skin examination three or more times during the past 4 months, while 37% (*n* = 19) reported self‐skin examination three or more times at the 4‐month follow‐up (*p* 0.63). The increase in reported self‐skin examination was compared between the two groups A: 51% versus group B: 17.6%, (*p* 0.001). The results suggest that a brief nurse‐delivered intervention is effective at increasing patient adherence with self‐skin examination. Utilising digital photographs as an adjunct to screening appeared to increase patient adherence to performing self‐skin examination. Cost effectiveness: not mentioned. Value added care for patients: authors concluded that ‘patient acceptance of the photographs was high’ but did not specify the method used to determine this conclusion. Value added care for service users: not mentioned.
Strengths and weaknesses	Strengths: this paper makes a valuable contribution by demonstrating that nurse‐led care can effectively meet healthcare needs, delivering services traditionally provided by physicians and offering comprehensive care from assessment to discharge when managed by highly trained, qualified and competent nurses. Brief service user feedback indicated that nurse‐led care was acceptable to patients. Cost effectiveness was mentioned but a full cost comparison was not conducted. Weaknesses: the sample size was small because of the 12‐week time frame. No direct comparisons were made to physicians. Patient and public involvement strategy prior to or during the study: was not mentioned.	Strengths: this paper adds valuable evidence demonstrating that an independent nurse‐led service for managing benign eyelid lesions is fit for purpose. For patients meeting the criteria for nurse‐led treatment, care was effectively provided by nurses, replacing physician‐led care and encompassing comprehensive management. The study examined six years of data, providing a robust sample size. Participant demographics were included and were comparable in both groups. Cost effectiveness of nurse‐led care was demonstrated. Weaknesses: no service user feedback was collected. Patient and public involvement strategy prior to or during the study: was not mentioned.	Strengths: this study shows that photodynamic therapy treatment was fit for purpose when delivered by the same nurse and using the same equipment in either a community or hospital setting. Brief service user feedback was collected, and patients favoured community care. Weaknesses: while some costs were measured, a comprehensive cost comparison and analysis were not conducted. Additionally, the study did not address whether patients requiring local anaesthetic for treatment were accommodated or if the nurse was a qualified prescriber. The sample size was small. Demographics were incompletely described or not collected and was limited to the average age of participants. Patient and public involvement strategy prior to or during the study: was not mentioned.	Strengths: this paper shows that nurses delivered nurse‐led technology supported care that was fit for purpose. The nurses facilitated dermatologist assessments on patients with skin lesions. Weaknesses: the lesions for which the general practitioners did not provide images or images were deemed inadequate were seen in the virtual lesion clinic and therefore not a randomised comparison. General practitioner triage was subject to selection bias. Service user feedback was not mentioned. Patient and public involvement strategy prior to or during the study: was not mentioned.	Strengths: this paper adds value because it demonstrates that a nurse‐led model can be cost effective. According to the financial analysis conducted, nurses time cost less than a physician. Some information about service users' satisfaction and acceptability was collected and patients rated the virtual lesion clinic higher on all surveyed items. Weaknesses: only age and gender of participants was described. Dermatologist feedback could have revealed whether the virtual lesion clinic impacted positively on workload. Cost to patients for travelling etc. was not measured. Patient and public involvement strategy prior to or during the study: was not mentioned.	Strengths: this study supports the use of nurse‐led patient education. The educational interventional had multiple components. Participant demographics were well described. Weaknesses: the method of randomisation was not described. There was a short follow up of 4 months to measure adherence to skin self‐examination. This study was limited by the selected study groups, which were composed of patients at high risk for melanoma based on the presence of five or more dysplastic nevi and not representative of all patients that develop melanoma. The sample size for this study was small. As highlighted by the authors, the study was conducted in an experimental, highly controlled situation and participants were deemed highly motivated due to having already had a melanoma. Service user feedback was not gathered or reported in the study. While the authors stated that participants' acceptance of photographs was high, they did not explain the methodology or evidence supporting this conclusion. Patient and public involvement strategy prior to or during the study: was not mentioned.

#### 2.4.5 Data Synthesis

To ensure a rigorous and transparent narrative synthesis while minimising bias from subjective judgements, the review was guided by the four key elements outlined by Popay et al. (2006). An initial framework was developed to categorise and understand the functioning of nurse‐led models, including whether they replace or complement physician care, their effectiveness, the populations that benefit most and the settings in which they are most useful. The selected studies were tabulated to identify key similarities and differences, translating patterns into themes and exploring relationships in the data. An iterative process was used to reassess each paper and refine the synthesis, ultimately constructing a narrative that directly addressed the research questions of the review.

#### 2.4.6 Data Extraction

The principal reviewer (1) compiled data from the six studies into a Microsoft Excel spreadsheet, categorising the results in Table 3. For accuracy and consistency, two other reviewers (1 and 4) checked the data. Categories included geographical location, setting, data collection method, nurse‐led model type and nurse designation. Key themes such as full skin examination, treatment, health education, care equivalence to physicians, cost‐effectiveness, service user feedback and value‐added care for patients were identified. A summary of the demographics is presented in Table 4.

**TABLE 4:** Summary of the demographics of the participants of the included studies.

Paper	Type of study	Age of patients	Female patients	Male patients	Ethnicity of patients	Education of patients
Jones, C. and Mullen, L. A service evaluation of a nurse consultant‐led basal cell carcinoma clinic. 2014.	Mixed methods (qualitative and quantitative)	The age range of patients was from 25 to 94 years Mean age was 65 years (standard deviation =14.54 years) 33.9% (*n* = 40) of patients were in the 66–75 age group 24.4% (*n* = 29) were under the age of 55 years	61 women	57 men	Not mentioned	Not mentioned
Mohite, A. A., Johnson, A., Rathore, D. S., Bhandari, K., Crossman, R., Mehta, P. and Ahluwalia, H. S. Accuracy of clinical diagnosis of benign eyelid lesions: Is a dedicated nurse‐led service safe and effective? 2016.	Quantitative	The mean age at the time of excision was 56 years	56.5% (*n* = 266) females	43.5% (*n* = 205) males	Caucasian patients totalled: 85.6% (*n* = 402/471) Indian origin: 8.7% (*n* = 41/471) Black or Afro‐Caribbean: 1.7% (*n* = 8/471) Chinese or Oriental: 0.4% (*n* = 2/471) Mixed ethnicity: 0.6% (*n* = 3/471) Not specified: 3.2% (*n* = 15/471)	Not mentioned
Clayton, T. H., Tait, J., Whitehurst, C., and Yates, V. M. Photodynamic therapy for superficial basal cell carcinoma and Bowen's disease. 2006.	Quantitative	The average age of patients was 71 years	Not mentioned	Not mentioned	Not mentioned	Not mentioned
Jones, L., Jameson, M., and Oakley, A. Remote Skin Cancer Diagnosis: Adding Images to Electronic Referrals Is More Efficient Than Waitlisting for a Nurse‐Led Imaging Clinic. 2021.	Quantitative	Suspected skin cancer cohort: Mean age of 61 years (SD: 19.2) Matched suspected skin cancer pathway cohort: 55 years (SD: 21.0) 2016 virtual lesion clinic cohort: 55 years (SD: 21.0) 2020 virtual lesion clinic cohort: 59 years (SD: 16.1) Range for all groups 0 to 90+ years	Suspected skin cancer cohort: Females: 56% (*n* = 738) Matched suspected skin cancer pathway cohort: Females: 64% (*n* = 309) 2020 virtual lesion clinic cohort: Females: 59% (*n* = 64) 2016 virtual lesion clinic cohort: Females: 64% (*n* = 254)	Suspected skin cancer cohort: Males: 44% (*n* = 569) Matched suspected skin cancer pathway cohort: Males: 36% (*n* = 172). 2020 virtual lesion clinic cohort: Males: 41% (*n* = 44) 2016 virtual lesion clinic cohort: Males: 37% (*n* = 146)	Suspected skin cancer cohort: Caucasian New Zealand European: 84% (*n* = 1096) Māori ethnicity: 6% (*n* = 73) Suspected skin cancer matched cohort: Caucasian New Zealand European: 78% (*n* = 378) Māori ethnicity: 6% (*n* = 30) 2020 virtual lesion clinic cohort: Caucasian New Zealand European: 74% (*n* = 80) Māori ethnicity: 11% (*n* = 12) 2016 virtual lesion clinic cohort: Caucasian New Zealand European: 79% (*n* = 317) Māori ethnicity: 7% (*n* = 26)	Not mentioned
Lim. D., Oakley., A. M. and Rademaker, M. Better, sooner, more convenient: a successful teledermoscopy service. 2012.	Quantitative	Face‐to‐face group: 62.7 years (15–94) Virtual lesion clinic: 52.5 years (2–89)	Females within face‐to‐face group: 64 (*n* = 64) Females within virtual lesion clinic: 61% (*n* = 122)	Males within face‐to‐face group: 36% (*n* = 36) Males within virtual lesion clinic: 39% (*n* = 78)	Not mentioned	Not mentioned
Oliveria, S. A., Dusza, S. W., Phelan, D. L., Ostroff, J. S., Berwick, M. and Halpern, A. C. Patient adherence to skin self‐examination. effect of nurse intervention with photographs. 2004.	Quantitative	Age range of participants > 20 to ≥ 60 years Mean age of participants in years: Males: 43.4 years (SD = 13.01) Females: 37.9 years (SD = 10.4)	Group A females: 59.2% (*n* = 29) Group B females: 66.7% (*n* = 34)	Group A males: 40.8% (*n* = 20) Group B males: 33.3% (*n* = 17)	White non‐Hispanic: Group A: 97.9% (*n* = 48) Group B: 98% (*n* = 50) Unknown: Group A: 2.1% (*n* = 1) Group B: 2% (*n* = 1)	High school or less: Group A: 0% (*n* = 0) Group B: 3.9% (*n* = 2) Partial college/standard college/University graduate: Group A: 59.2% (*n* = 27) Group B: 60.8% (*n* = 31) Graduate degree or professional training: Group A: 40.8% (*n* = 22) Group B: 35.3% (*n* = 18)

#### 2.4.7 Outcomes and Prioritisation

Outcomes were reported as presented in the six individual studies, without pre‐determination prior to the literature search. As key themes emerged, other study findings were categorised according to outcome type. No specific outcomes were prioritised, ensuring a thorough review of the available evidence.

## 3 Findings

**FIGURE 1:**
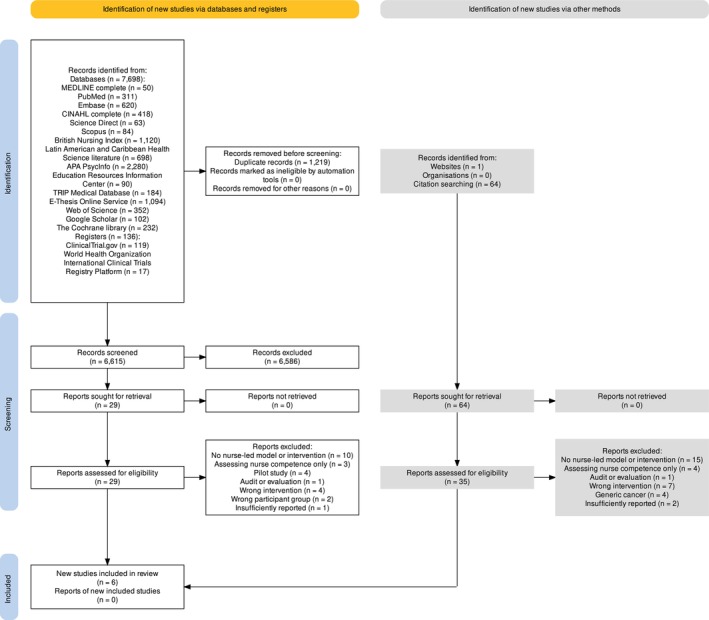
PRISMA Flow Diagram illustrating the process of study selection, including identification, screening, eligibility assessment and inclusion/exclusion criteria used in the review.

Six studies (Clayton et al. 2006; Jones et al. 2021; Jones and Mullen 2014; Lim et al. 2012; Mohite et al. 2016; Oliveria et al. 2004) met the eligibility criteria. The PRISMA flow diagram (Figure 1) outlines the search results at each stage of the screening process. Information sources included databases (*n* = 7698), registries (*n* = 136), websites (*n* = 1), organisations (*n* = 0), and forward and backward citation searches (*n* = 64). From a total of 7899 hits, 1219 duplicates were removed, leaving 6680 records for title and abstract screening. Of these, 93 reports were reviewed in full text, and six studies ultimately met the inclusion criteria (see Appendix S4 for the reference list of articles meeting the inclusion criteria).

Four studies were identified through database and registry searching, and two were located via backward citation searching. The six studies reported outcomes on a total of 3325 patients in three different countries: England, New Zealand and the United States. The characteristics of these studies have been summarised, including the study design, type of nurse‐led models, comparisons to physician‐led care (where available), outcome measures (Table 3) and participant demographics (Table 4). All elements of this review were conducted with a minimum of two reviewers to ensure accuracy and reliability.

### 3.1 Studies Reporting Assessment, Treatment and/or Education

All six studies described either a nurse‐led service delivery model (Clayton et al. 2006; Lim et al. 2012; Jones and Mullen 2014; Mohite et al. 2016; Jones, Jameson, and Oakley 2021) or intervention (Oliveria et al. 2004) delivered partially or entirely by registered nurses. Four studies involved collaboration with secondary care dermatologists (Clayton et al. 2006; Jones, Jameson, and Oakley 2021; Lim, Oakley, and Rademaker 2012; Oliveria et al. 2004), and one study involved an ophthalmologist (Mohite et al. 2016). One study by Jones and Mullen (2014) described a consultant nurse‐led service within secondary care. See Table 5 for a summary of the nurse‐led model types.

**TABLE 5:** Summary of the types of nurse‐led models or interventions.

Articles number	Article 1	Article 2	Article 3	Article 4	Articles 5	Article 6
Author and title	Jones, C. and Mullen, L. A service evaluation of a nurse consultant led basal cell carcinoma clinic.	Mohite, A. A., Johnson, A., Rathore, D. S., Bhandari, K., Crossman, R., Mehta, P. and Ahluwalia, H. S. Accuracy of clinical diagnosis of benign eyelid lesions: Is a dedicated nurse‐led service safe and effective?	Clayton, T. H., Tait, J., Whitehurst, C., and Yates, V. M. Photodynamic therapy for superficial basal cell carcinoma and Bowen's disease.	Jones, L., Jameson, M., and Oakley, A. Remote Skin Cancer Diagnosis: Adding Images to Electronic Referrals Is More Efficient Than Waitlisting for a Nurse‐Led Imaging Clinic.	Lim. D., Oakley., A. M. and Rademaker, M. Better, sooner, more convenient: a successful teledermoscopy service.	Oliveria, S. A., Dusza, S. W., Phelan, D. L., Ostroff, J. S., Berwick, M. and Halpern, A. C. Patient adherence to skin self‐examination. effect of nurse intervention with photographs.
Summary of types of nurse‐led models and interventions within the included studies	A service evaluation of a nurse consultant‐led basal cell carcinoma clinic delivered within a National Health Service dermatology service. The role also included skin surgery. Patients with lesions suspicious of a basal cell carcinoma were referred into secondary care by their general practitioner for assessment. The nurse consultant provided care from initial assessment to discharge.	An oculo‐plastic clinical nurse specialist‐led benign eyelid lesion clinic. All suspected benign lesions were triaged and diagnosed and then excised by the same clinical nurse specialist. New lesions referred to the oculoplastic service were either directed to the doctor‐led clinic or deemed suitable for the nurse‐led clinic by either the consultant or the clinical nurse specialist who received and vetted referrals separately. Cases were directed away from the nurse‐led clinic if there was a suspicion of malignancy, lid margin involvement or deemed too large for suitable surgical excision.	A dermatology clinical nurse specialist‐led photodynamic therapy clinic comparing standard hospital‐based care with 5‐aminolaevulinic acid cream and light administration to community‐based care where the cream is applied at home, and light therapy is delivered at a local health centre.	A specially trained nurse melanographer‐led virtual lesion clinic was compared to a general practitioner‐led imaging and electronic referral service. In the nurse‐led model, primary care doctors refer patients to a community teledermoscopy clinic at one of three locations, where the nurse photographs the lesion(s) and refers them to a dermatologist in secondary care for assessment. In the general practitioner‐led model, doctors use a suspected skin cancer pathway, attaching regional, close‐up, and dermoscopic images along with the patient history and electronically referring them to a dermatologist for evaluation.	A specially trained nurse melanographer‐led virtual lesion clinic involved taking a standardised history of the lesion(s) of concern along with relevant medical background. Using specially adapted cameras, regional, anatomic, macroscopic, and dermoscopic photographs of the skin lesions were captured and sent to a dermatologist for management decisions. These decisions could include discharge, follow‐up imaging to monitor changes, or referral for treatment. Treatment options included management by the referring general practitioner, the nurse clinic, or a hospital‐based dermatologist or plastic surgeon.	The effect of a dermatology‐trained nurse‐led intervention with photographs on patient adherence to skin self‐examination was assessed. At baseline, a physician examined each participant to document the number of moles and dysplastic nevi, and melanocytic lesions suggestive of early melanoma were removed. Following the examination and completion of a baseline questionnaire, participants attended an educational session led by the nurse. The participants were randomised to either intervention A or B. Intervention A was a teaching intervention (physician and nurse education modules) with photo book (personal whole‐body photographs in the form of a booklet, with nurse instruction on how to use the photographs as an adjunct to self‐skin examination). Or intervention B, the teaching intervention with no photo book and instead a written pamphlet on how to perform self‐skin examination and how to record moles in a diary format.

#### 3.1.1 Assessments and Full Skin Examinations

One study conducted in England (Jones and Mullen 2014) reported that a nurse consultant performed full skin examinations on each patient, detecting additional, unsuspected lesions in 29.1% of patients (*n* = 34). In contrast, Oliveria et al. (2004) in the United States reported that dermatologists conducted baseline skin examinations, with no assessments being performed by nurses. In New Zealand, Lim, Oakley, and Rademaker (2012) and Jones, Jameson, and Oakley (2021) indicated that dermatologists performed skin examinations if participants were referred to secondary care. However, Jones, Jameson, and Oakley (2021) noted that clinical nurse specialists identified 107 incidental lesions in community imaging clinics, suggesting that nurses performed skin examinations, although specific details of these examinations were not provided. Lim, Oakley, and Rademaker (2012) pointed out that a limitation of the virtual lesion clinic was the absence of full‐body examinations.

In two studies conducted in England (Clayton et al. 2006; Mohite et al. 2016), full skin examinations were not mentioned. Clayton et al. (2006) described nurses administering photodynamic therapy under the direction of dermatologists, who later assessed treatment success. Mohite et al. (2016) described a study within an ophthalmology service where clinical nurse specialists assessed suspected benign eyelid lesions and following assessment against specific criteria, referred patients to appropriate surgeons. In some cases, they performed surgical removal of benign lesions themselves, as appropriate.

#### 3.1.2 Treatment

The nurse consultant determined that 83 (98%) out of 118 patients assessed required surgical intervention for their lesions (Jones and Mullen 2014). Of these, the nurse consultant operated on 57 (43%) patients, while 17 (14%) received surgical treatment from an advanced nurse practitioner. For patients not requiring surgical intervention, the nurse consultant administered treatment. A total of 35 patients (41%) received cryotherapy (freezing spray treatment) or topical treatment with 5‐fluorouracil or imiquimod cream, all of which work by destroying the damaged cells (British Association of Dermatologists 2018, 2022, 2024).

In England, two nurse‐led models were described: one involving hospital‐based surgical removal of benign eyelid lesions (Mohite et al. 2016), and the other providing photodynamic therapy in both hospital and community settings (Clayton et al. 2006). Additionally, a study by Lim, Oakley, and Rademaker (2012) in New Zealand reported that nurses provided treatments, including cryotherapy and unspecified topical therapies; however, it was unclear whether they also performed minor surgical procedures. The study excluded surgery costs from the financial analysis, as these costs would be similar whether patients were diagnosed at the virtual lesion clinic or in face‐to‐face clinics. In the study by Jones, Jameson, and Oakley (2021) conducted in New Zealand, treatments were mentioned but it was unclear whether these were performed by nurses.

#### 3.1.3 Education

Health education was only noted in two studies (Jones and Mullen 2014; Oliveria et al. 2004). Oliveria et al. (2004) described a nurse‐led educational intervention, previously outlined in a pilot study (Phelan et al. 2003), where nurses presented a 3‐minute video on skin self‐examination. The video promoted early detection by systematically guiding patients through each step using imagery and was followed by a question‐and‐answer session. In contrast, Jones and Mullen (2014) mentioned health education delivery but did not provide specific details.

### 3.2 Studies Reporting Effectiveness of Nurse‐Led Care Compared to Physician‐Led Care

#### 3.2.1 Effectiveness

Overall, the studies reviewed consistently demonstrated that nurse‐led services were fit for purpose (Jones and Mullen 2014; Mohite et al. 2016; Clayton et al. 2006; Jones, Jameson, and Oakley 2021; Lim, Oakley, and Rademaker 2012; Oliveria et al. 2004). A high‐quality evaluation of a nurse consultant‐led basal cell carcinoma clinic in England found that nurses delivered comprehensive care to 89% (*n* = 105) of patients without dermatologist input (Jones and Mullen 2014). Most patients underwent complete excision of their lesion with no follow‐up required. Nurses also identified additional lesions during full skin examinations, though no direct comparison with physicians' diagnostic accuracy was made. This study was notable for its clear inclusion criteria and reliable measurement methods, despite some limitations in demographic reporting and statistical analysis.

A retrospective comparative study conducted within a secondary care ophthalmology service in England examined outcomes between nurse‐led and physician‐led services for skin lesion management (Mohite et al. 2016). The study had clear inclusion criteria, comparable demographic reporting and appropriate statistical analyses. No significant differences in patient demographics between the two services (*p > 0.05*) were identified. Both services had similar distributions of benign lesions confirmed by histology, with diagnostic accuracy rates of 80% (267/332) for nurse‐led services and 79.6% (210/264) for physician‐led services. Additionally, the missed malignancy rates were low and comparable between the two groups, at 1.5% (*n* = 4) for physicians and 1.1% (*n* = 4) for nurses (*p > 0.05*).

A small, randomised study compared nurse‐led photodynamic therapy in a hospital to community‐based therapy, with outcomes assessed by a dermatologist (Clayton et al. 2006). At six months, results were similar between both groups. While the study was rated as moderate quality for demonstrating clear cause‐and‐effect and having sufficient initial follow‐up, limitations were noted. These included inadequate randomisation, where group allocation was based on lesion order, with the first lesions treated in secondary care, followed by subsequent ones in primary care. The study also lacked detailed statistical analysis and had insufficient outcome measures, likely due to a lack of long‐term follow‐up and a small sample size.

A retrospective study (Jones, Jameson, and Oakley 2021) compared the effectiveness of a 2020 suspected skin cancer referral pathway with nurse‐led virtual lesion clinics from 2016 and 2020. In both services, patients with suspicious skin lesions were assessed virtually by a dermatologist using regional, close‐up and dermoscopic images. In the virtual lesion clinics, specialist nurse melanographers captured images of lesions in a community setting, which were then remotely evaluated by a dermatologist (Jones, Jameson, and Oakley 2021). The study identified a statistically significant reduction in the median time from referral to advice (*p < 0.001*), with the suspected skin cancer pathway taking four days compared to 42 and 50 days for the virtual lesion clinics in 2020 and 2016, respectively. Dermatologists recommended ‘no further action’ for 56% (*n* = 298) of lesions in the suspected skin cancer pathway cohort, 57% (*n* = 157) in the 2020 virtual lesion clinic cohort, and 69% (*n* = 471) in the 2016 cohort. Clinic nurse specialists working remotely provided high‐quality images using standard cameras, though 5% (*n* = 71) of lesions in the suspected skin cancer pathway were undiagnosable due to poor image quality. The study, assessed as moderate quality, had valid outcome measurements but identified confounding factors. Both groups were recruited from the same population, though potential selection bias existed due to general practitioner triaging for group allocation.

Lim, Oakley, and Rademaker (2012) compared patient flow between a community‐based nurse‐led virtual lesion clinic and a tertiary hospital face‐to‐face dermatology clinic. The mean waiting time for an appointment in the face‐to‐face clinic was 114 days, compared to 39 days for the virtual lesion clinic, representing a 66% reduction in waiting time for the latter. At the start of the study, the mean waiting time for the virtual clinic was 37 days, while the face‐to‐face group's mean waiting time was 66 days, peaking at a mean of 138 days. Dermatologists remotely assessed 383 lesions in the virtual clinic, compared to 210 in the face‐to‐face group. Treatment was recommended for fewer patients in the virtual clinic 36.5% (*n* = 73) compared to the face‐to‐face clinic 60% (*n* = 60), and 59% (*n* = 117) of virtual clinic patients were discharged. The study was rated as low quality due to differences in group recruitment, unclear measurement validity, unidentified confounding factors, incomplete follow‐up and insufficient strategies to address loss to follow‐up. Despite these limitations, the outcome measurements were still deemed valid and reliable.

A case–control study (Oliveria et al. 2004) revealed increased adherence to skin self‐examination at the 4‐month follow‐up for both interventions, with the photobook intervention showing superior improvement. The study had a participation rate of 95% (*n* = 100/105). In Group A (teaching intervention with a photobook) and Group B (teaching intervention without a photobook), 86% (*n* = 42) and 84% (*n* = 43) respectively completed the 4‐month follow‐up. In Group A, 51% (*n* = 25) showed improved skin self‐examination at the 4‐month follow‐up, compared to 17.6% (*n* = 14) in Group B. The photobook intervention had a statistically significant effect (*p < 0.001*) on adherence to skin self‐examination. Additionally, 10.2% (*n* = 5) in Group A reported skin examination three or more times at baseline, increasing to 61.2% (*n* = 30) at the 4‐month follow‐up (*p = 0.039*). In Group B, nearly 20% (*n* = 10) reported skin examination three or more times at baseline, rising to 37% (*n* = 19) at the 4‐month follow‐up (*p = 0.63*). The increase was 51% in Group A and 17.6% in Group B, a statistically significant difference (*p < 0.001*). The study by Oliveria et al. (2004) was rated as moderate quality, with strengths in case–control matching and valid outcome assessments. However, there was a lack of clarity regarding confounding factors, and the short exposure period limited the ability to assess the long‐term effects of the photobook on self‐skin examination monitoring.

#### 3.2.2 Service User Feedback

In two studies service user feedback was absent (Jones, Jameson, and Oakley 2021; Mohite et al. 2016). Oliveria et al. (2004) mentioned that patient acceptance of the photographs was high but did not specify the method for measuring this acceptance. In the study by Jones and Mullen (2014), qualitative patient testimonies were collected by a clinical nurse specialist via telephone and follow‐up clinics. Patient testimonies included feelings of reassurance throughout treatment, satisfaction with scarring, appreciation for the prompt identification and treatment of serious skin cancers, shorter waiting times relative to prior experiences and increased confidence in treatments due to thorough explanations. Key themes from the feedback highlighted a lack of clarity surrounding the nurse consultant's role. Despite this, patients shared positive views about the care they received, often expressing pleasant surprise at the comprehensive and autonomous care provided by the nurse consultant (Jones and Mullen 2014).

A study by Clayton et al. (2006) compared hospital‐administered photodynamic therapy with community‐based photodynamic therapy, involving 25 patients in each group. The average age of patients receiving treatment was 71 years. The study highlighted logistical challenges specific to hospital‐based care, such as patients needing to cancel entire‐day commitments for treatment and relying on relatives for transportation. Community‐treated patients stayed home during the cream absorption phase (5‐aminolaevulinic acid), leading to shorter treatment times at the health centre. All 25 completed therapy within 1–2 h, with 88% finishing in under an hour. In contrast, hospital‐treated patients experienced longer durations due to attending for photosensitiser application, with 8% spending over 6 h, 64% between 3 and 6 h, and 28% finishing in under 3 h. Patients preferred community‐based treatment for its convenience and accessibility, with 96% (*n* = 24) of community‐treated patients and 52% (*n* = 13) of hospital‐treated patients favouring the local health centre.

The survey data from Lim, Oakley, and Rademaker (2012) compared service user perceptions of the nurse‐led virtual lesion clinics and the face‐to‐face physician‐led clinics, using a rating scale from 1 (poor) to 5 (excellent) across several key elements. Respondents rated the waiting time for appointments significantly higher at the virtual lesion clinic (4.0) compared to the face‐to‐face clinic (2.9), indicating shorter and more favourable waiting times. Similarly, convenience and waiting times at the clinic were perceived more favourably at the virtual lesion clinic (4.1–4.7) than at the face‐to‐face clinic (3.3–3.8). Service users also reported higher satisfaction with the time spent with health professionals and the quality of explanations provided at the virtual lesion clinic (4.5) compared to the face‐to‐face clinic (4.1). The overall experience of the service was rated significantly higher at the nurse‐led virtual lesion clinic (4.5) compared to the physician‐led face‐to‐face clinic (3.8), suggesting that users of the virtual clinic reported a more positive experience.

#### 3.2.3 Cost Effectiveness

Cost‐effectiveness was not addressed in two of the six studies (Jones, Jameson, and Oakley 2021; Oliveria et al. 2004). The Jones and Mullen (2014) study on a nurse‐led model carried out in England found it to be comparable to a medical consultant‐led service, delivering equivalent care while requiring less funding. Although they claimed the service was cost‐effective, no financial data or analysis was provided. Another advantage of the nurse‐led model was that it created capacity for additional physician‐led clinics.

In a National Health Service ophthalmology service in England, which involved examining suspicious skin lesions, an experienced nurse was reported to be approximately 55% less costly than a consultant or senior staff grade per minor operation list, resulting in an 18%–23% cost saving per 8‐patient lesion list (Mohite et al. 2016). The study suggested that employing nurses for minor operation lists could free up physician time for complex cases; however, the authors did not provide detailed cost analyses or comparative data on clinic efficiency.

Clayton et al. (2006) provided a cost breakdown for a single photodynamic therapy treatment within the National Health Service in England, detailing expenses for light administration (£80), sensitising agent (£24 per lesion), nursing (£10/h) and consumables (£5). Costs were lower in the primary care group due to reduced transport expenses, as 10 out of 25 hospital‐treated patients required an ambulance (£75 per trip). Some patients relied on friends or family for transport when ambulances were unavailable.

Significant savings were achieved through virtual lesion clinics, as reported by Lim, Oakley, and Rademaker (2012). The financial analysis included total patient interactions, diagnostic visits and follow‐up nurse treatment clinics. Costings from the District Health Board's business unit in New Zealand revealed a contracted fee per patient for the virtual clinic. Nurse‐led treatments like cryotherapy and topical therapy saved $42.00 per patient, reducing costs by 14%.

### 3.3 Studies Reporting Value‐Added Care in Nurse‐Led Models Compared to Physician‐Led Care (Standard Care)

Value‐added care in nurse‐led skin cancer management refers to services provided in addition to standard care equivalence, such as health education and accessible, community‐based care. Mohite et al. (2016) compared diagnostic accuracy between clinical nurse specialists and physicians but did not address value‐added care. The remaining studies (Clayton et al. 2006; Jones, Jameson, and Oakley 2021; Jones and Mullen 2014; Lim, Oakley, and Rademaker 2012; Oliveria et al. 2004) did not directly compare nurse‐led to physician‐led care, limiting assessment of value‐added care. However, nurse‐led models effectively delivered the required care. Benefits included reduced travel times (Clayton et al. 2006; Jones, Jameson, and Oakley 2021; Lim, Oakley, and Rademaker 2012), shorter waiting times (Jones and Mullen 2014; Lim, Oakley, and Rademaker 2012), shorter treatment times (Clayton et al. 2006), increased access (Clayton et al. 2006; Jones and Mullen 2014; Lim, Oakley, and Rademaker 2012), health education (Jones and Mullen 2014) and reduced patient costs (Clayton et al. 2006; Lim, Oakley, and Rademaker 2012).

## 4 Discussion

This review evaluates international nurse‐led models for skin cancer assessment, treatment and education, comparing their effectiveness to standard care. The heterogeneity and variability of data across studies precluded a quantitative synthesis. Consequently, a narrative synthesis was undertaken, potentially limiting the generalisability of the findings and highlighting the need for more standardised evaluation frameworks. Courtenay and Carey (2007) highlighted the significant benefits of nurse interventions in dermatology, including reduced condition severity, improved use of topical therapies, faster access to care, fewer referrals and increased patient knowledge. However, they also noted that some primary care nurses lack confidence, and their educational needs are often unmet. Furthermore, despite generally positive findings, methodological weaknesses and under‐researched areas, particularly cost‐effectiveness and nurse prescribing, point to the need for further rigorous evaluation. The van Os‐Medendorp et al. (2020) review details the role of specialised dermatology nurses in managing moderate to severe atopic dermatitis, emphasising their specific contributions to education, support and treatment adherence.

The National Institute for Health and Care Excellence (2024) guideline for skin cancer recommends that patients with melanoma, high‐risk squamous cell carcinoma, or rare skin cancers have access to a clinical nurse specialist. These specialists manage chronic and complex conditions (Tracy et al. 2020). Nurse consultants, the most advanced role within clinical nursing practice, are involved in expert practice, leadership, education, training, service development and research (Redwood, Carr, and Graham 2005). Jones and Mullen (2014) reported positive patient feedback regarding care from a nurse consultant, though issues with role clarity were identified. However, this conclusion is drawn from a single study. The ‘Getting It Right First Time’ dermatology report for England found that 11% of National Health Service trusts have a dermatology nurse consultant in post, 76% offer nurse‐led skin cancer clinics, and 23% provide nurse‐led clinics specifically for new skin cancer patients (GIRFT 2021). A census of skin cancer specialist nurses in the United Kingdom found that rising referrals and increased demand have led to the expansion of nurse‐led clinics, often operating with reduced consultant oversight (Rammanohar et al. 2023).

Clinical nurse specialists perform advanced tasks, including independent non‐medical prescribing for specific groups, ordering investigations, and drug monitoring (BDNG 2023). In contrast, advanced nurse practitioners and nurse consultants assume higher‐level responsibilities, such as diagnostics and more comprehensive prescribing (*Health Education England* 2017). Health Education England's framework for advanced clinical practice, seeks to promote national consistency in key role elements (*Health Education England* 2017). Despite progress, Hardy (2021) highlights ongoing inconsistencies in the descriptions and titles of advanced nursing roles across different healthcare settings and professional groups. In the United States, the National Council of State Boards of Nursing (2024) regulates Advanced Practice Registered Nurses, ensuring they are qualified to assess, diagnose, manage, order tests, and prescribe medications through advanced education and certification. Meanwhile, the United Kingdom's Nursing and Midwifery Council is currently reviewing the need for additional regulation to standardise advanced nursing practice (NMC 2024).

Poghosyan and Maier (2022) emphasise the increasing evidence base demonstrating the effectiveness and quality of advanced practice nursing across multiple stages of implementation and clinical specialties. They point out that while countries like Norway are in the early phases of adopting advanced practice roles, nations such as England and the United States have more established systems, showcasing the successful integration of these roles into healthcare. The International Council of Nurses (ICN 2020) guidelines on Advanced Practice Nursing stress the importance of a shared understanding of roles and responsibilities among key stakeholders, including the public, governments, healthcare professionals, policymakers, educators and the nursing profession. These guidelines aim to establish policies, frameworks and strategies to ensure consistency and clarity in Advanced Practice Nursing roles worldwide, helping Advanced Practice Registered Nurses contribute effectively to healthcare needs (Bryant‐Lukosius et al. 2017; Carryer et al. 2018). The International Council of Nurses also emphasises the dynamic nature of advanced practice nursing, encouraging its continuous development through education, regulation and practice adjustments to address evolving healthcare demands (International Council of Nurses 2020).

With global skin cancer cases projected to increase (Hasan et al. 2023; Zhang et al. 2021), there is an urgent need to strengthen public health education and expand care capacity. A pilot study in Australia, the ‘Skin Cancer Assessment Remote Service’, focused on personalised education in skin cancer detection, self‐examination, sun protection and the development of nursing skills. It also included nurse‐led assessments using dermatoscopy (Christensen 2019). In the initial pilot, 54 patients were screened; 20% (*n* = 11) were identified as high risk and 79% (*n* = 43) as medium risk for melanoma and non‐melanoma skin cancer. Notably, 49% (*n* = 26) of these patients had never been screened before. Nurses successfully identified and treated 6 (*n* = 11%) malignant melanomas, 8 (*n* = 15%) squamous cell carcinomas and 7 (*n* = 13%) basal cell carcinomas.

Studies confirm that nurses demonstrate high diagnostic accuracy when assessing referred skin lesions (Jones and Mullen 2014; Mohite et al. 2016) and identified previously unsuspected lesions, such as invasive melanoma and squamous cell carcinoma (Jones and Mullen 2014; Jones, Jameson, and Oakley 2021). Mohite et al. (2016) emphasised the critical role of nurse training in achieving accurate diagnoses, recommending that clinicians pre‐vet referrals to ensure appropriate cases for nurse‐led services. With pre‐vetting, nurse‐led services were found to be collaborative, cost‐effective and comparable to physician‐led care. Nurses demonstrated high diagnostic accuracy, particularly for benign lesions, and effectively managed smaller, more complex cases, highlighting their level of expertise when provided with adequate training. However, the lack of direct comparisons between nurse‐led and dermatologist‐led care represents a significant gap. Future research must address this limitation to provide a clearer understanding of their relative effectiveness and cost‐efficiency, which would better inform healthcare policy and practice.

Financial analyses have demonstrated significant cost savings for nurse‐led virtual lesion clinics compared to face‐to‐face dermatologist appointments (Lim, Oakley, and Rademaker 2012). Both Jones and Mullen (2014) and Mohite et al. (2016) found nurse‐led care to be cost‐effective and comparable to consultant‐led services. Mohite et al. (2016) reported approximately 50% savings per nurse‐led surgical list; however, the details of the cost calculation and included expenses were not provided. Additionally, nurse‐led care helped free up consultant physician time for other priority tasks in both studies (Jones and Mullen 2014; Mohite et al. 2016). Nurse‐led care increased access to skin cancer services in studies by Clayton et al. (2006), Jones and Mullen (2014) and Lim, Oakley, and Rademaker (2012), with Jones and Mullen (2014) noting that health education was also provided alongside standard care.

Several studies have highlighted other significant benefits of nurse‐led skin cancer care, including reduced travel time for patients (Clayton et al. 2006; Jones, Jameson, and Oakley 2021) and shorter waiting times for appointments (Jones and Mullen 2014; Lim, Oakley, and Rademaker 2012), enhancing both convenience and accessibility.

Nurse‐led, community‐based photodynamic therapy, administered locally, had treatment times of 1–2 h compared to the 3–6 h typically required when delivered in a hospital setting (Clayton et al. 2006). Nurse‐led care was found to increase access to care (Clayton et al. 2006; Jones and Mullen 2014; Lim, Oakley, and Rademaker 2012), provide additional health education (Jones and Mullen 2014), and reduced patient costs by offering care closer to home (Clayton et al. 2006; Lim, Oakley, and Rademaker 2012). Community outreach enhances accessibility, particularly for rural patients who face difficulties attending hospital appointments (Clayton et al. 2006). Nurse prescribing, a key element of advanced clinical practice (Health Education England 2017), is essential in treatment clinics to support the provision of comprehensive patient care.

Health education delivered to patients has been shown to encourage skin self‐examination (Oliveria et al. 2004), with females presenting more frequently with smaller lesions (Jones and Mullen 2014). For both sexes, family discussions about sun protection have been associated with increased engagement in sun protection behaviours (Manne et al. 2021). A systematic review and meta‐analysis on interventions promoting early skin cancer detection through skin self‐examination found that most studies targeted high‐risk individuals rather than the general population (Ersser et al. 2019). While some interventions demonstrated improvements in skin self‐examination activity and showed potential for facilitating early skin cancer detection, the limited quality of evidence restricted definitive conclusions regarding their clinical impact (Ersser et al. 2019). Oliveria et al. (2004) emphasised the importance of cues to action, such as nurse guidance and personalised photo books, in promoting behaviour change based on the health belief model (Rosenstock, Strecher, and Becker 1988).

Systematic self‐skin examination has been shown to reduce the incidence of thick lesions, morbidity and melanoma mortality, with visual aids, such as photographs, enhancing adherence. Nurse‐led models address critical gaps in skin cancer care by supporting physicians in care delivery, providing services that may otherwise be unavailable, ensuring equivalent care and offering value‐added benefits. However, significant evidence gaps persist, highlighting the need for comparative trials, cost analyses and long‐term evaluations to validate the efficacy and scalability of nurse‐led models across diverse settings.

### 4.1 Limitations

A key limitation is that only six studies met the inclusion criteria, narrowing the evidence base and limiting the findings' scope. This hinders definitive conclusions about the comparative effectiveness of nurse‐led and dermatologist‐led models, which may affect the generalisability of the results. However, this reflects the review's specific focus on studies addressing nurse‐led skin cancer care to ensure high relevance. An ophthalmology‐based study was also included due to its alignment with the review's objectives on lesion‐related care.

The varying quality of the studies, rated as low (*n* = 1), moderate (*n* = 3) and high (*n* = 2), introduces biases and weakens the robustness of the conclusions. Heterogeneity in aims, reporting and outcomes hindered comparison and synthesis, preventing meta‐analysis. The diversity of nurse‐led models and variations in outcome measures complicated comparisons with physician‐led models, limiting generalisable recommendations and highlighting the need for standardised frameworks to ensure consistent future comparisons.

The multidisciplinary approach in some models (Jones, Jameson, and Oakley 2021; Lim, Oakley, and Rademaker 2012) made it difficult to delineate the exact roles and contributions of nurses within each service. Notably, physicians were the first authors in four of the six studies (Clayton et al. 2006; Jones, Jameson, and Oakley 2021; Lim, Oakley, and Rademaker 2012; Mohite et al. 2016), and one study was authored by an epidemiologist (Oliveria et al. 2004). This may have contributed to an underrepresentation of the multifaceted components of nurse‐led models, including additional value‐added care for patients. In one study, it was unclear whether a full skin examination was provided by the nurse‐led service due to incomplete details (Jones, Jameson, and Oakley 2021).

Three studies had small sample sizes and short durations (Clayton et al. 2006; Jones and Mullen 2014; Oliveria et al. 2004). Notably, Oliveria et al. (2004) had a follow‐up period of only four months, which is significant given the focus on adherence to skin self‐examination. This raises concerns about the intervention's long‐term effectiveness, given that patients typically retain only 20% of medical information and forget 40%–80% shortly after medical encounters (Ley 1979; Richard, Glaser, and Lussier 2017; Street Jr. 2013; Sherlock and Brownie 2014).

Cost‐effectiveness was claimed in four studies (Clayton et al. 2006; Jones and Mullen 2014; Lim, Oakley, and Rademaker 2012; Mohite et al. 2016); however, only one study (Lim, Oakley, and Rademaker 2012) provided sufficient detail about costings through a financial analysis. This lack of transparency in the remaining studies limits the reliability of the cost‐effectiveness claims. To address this, it is crucial for nurses to describe and publish their nurse‐led models with detailed, rigorous evaluations, ensuring transparency and evidence‐based validation of their safety, efficiency and cost‐effectiveness.

## 5 Conclusion

The global shortage of dermatologists, driven by funding disparities and varying healthcare demands across countries, is expected to persist. This review demonstrates that nurse‐led care appears to be a safe, efficient and cost‐effective approach for managing specific aspects of the skin cancer care pathway under both direct and indirect physician supervision. Nurses have the potential to play a pivotal role in diagnostic services, treatment delivery and patient education on skin self‐examination, offering additional value‐added care to patients. However, further rigorous research is required to comprehensively evaluate these models.

While patient satisfaction with nurse‐led care is generally high, ambiguity surrounding role definitions remains a significant barrier to fully realising its potential. This highlights the urgent need for standardised, scalable and adaptable models tailored to local healthcare systems. Standardisation would enable nurse‐led care to support dermatologists in addressing high referral demands while ensuring the delivery of consistent and equitable care globally. Additionally, robust, trial‐based evaluations are critical to assess resource efficiency, cost‐effectiveness and the overall contribution of nurse‐led care to patient outcomes.

## Author Contributions

All authors, except Abdulrahman Shadeed, contributed to the initial conceptualisation of the study and the development of the study protocol in partnership with other collaborators. Leila Kattach identified relevant articles and independently screened them alongside Abdulrahman Shadeed. The final list of articles for inclusion was determined through consultation with Steven Ersser and Heidi Singleton. Quality appraisal of the included studies was jointly conducted by Leila Kattach and Heidi Singleton. Data extraction was carried out by Leila Kattach and subsequently reviewed by Heidi Singleton and Debbie Holley. All authors participated in data interpretation, manuscript revision and approved the final submitted version.

## Conflicts of Interest

The authors declare no conflicts of interest.

## Supporting information


Appendix S1.

Appendix S2.

Appendix S3.

Appendix S4.

## Data Availability

Data available in article's supporting Information.
