# Supplementary material for: Nurse‐Led Models of Service Delivery for Skin Cancer Detection: A Systematic Review
Source: J Adv Nurs. 2025 Apr 1;81(12):8432–56. doi: 10.1111/jan.16854 (PMC12623683; doi:10.1111/jan.16854)
Supplement: Supplementary file 2 — Appendix S2. [file JAN-81-8432-s003.docx]

**Supplementary document 2**

# **Search Strategy and Sources**

All initial searches were conducted starting from 19 June 2023. The information sources searched included electronic databases, search engines, webpages, and clinical trials registries. International dermatology nursing organisations were also contacted directly via email. The date that each information source was searched has been detailed in this document.

Figures in grey-shaded cells indicate the final search results included in the screening process.

**Please note that these searches were rerun on 10 September 2024, and no new articles met the inclusion criteria during the rerun.**

## **Summary Table of Results from All Information Sources**

| **Information Source**  **Number** | **Information Source Name** | **Number of Hits** |
| --- | --- | --- |
| 1 | Medline complete | 50 |
| 2 | Pubmed | 311 |
| 3 | EMBASE | 620 |
| 4 | CINAHL ultimate | 418 |
| 5 | Science direct | 63 |
| 6 | Scopus | 84 |
| 7 | British Nursing Index | 1120 |
| 8 | Latin American and Caribbean Health Sciences Literature (LILACS) | 698 |
| 9 | APA PsycInfo | 2280 |
| 10 | Education Resource Information Centre | 90 |
| 11 | TRIP | 184 |
| 12 | EThOs (E-Theses Online Service) | 1094 |
| 13 | Web of science | 352 |
| 14 | Google scholar | 102 |
| 15 | The Cochrane library which includes both [Cochrane Central Register of Controlled Trials](https://www.cochranelibrary.com/) (CENTRAL) and Cochrane Database of Systematic Reviews (CDSR) | 232 |
| 16 | Clinical trials. Gov | 119 |
| 17 | World Health Organization International Clinical Trials Registry Platform | 17 |
| 18 | Getting it right first time (website) | 1 |
| 19 | Organisations: British Dermatological Nursing Group, European Academy of Dermatology Nursing Taskforce Group, Australian Dermatology Nurses Association, Dermatology Nurses' Association (USA), and New Zealand Dermatology Nurses Society. | 0 |
| 20 | Citation searching | 64 |

### Search history from MEDLINE complete

This table shows the search performed on 20 February 2024

| **Search ID** | **Search Terms** | **Search Options** | **Results** |
| --- | --- | --- | --- |
| S1 | (MH "Skin Neoplasms") OR (MH "Neoplasms, Adnexal and Skin Appendage") | Limiters - Date of Publication: 19920101-; Human  Search modes - Boolean/Phrase | 98,173 |
| S2 | Skin cancer* | Limiters - Date of Publication: 19920101-; Human  Search modes - Boolean/Phrase | 101,000 |
| S3 | (Skin adj2 cancer*) | Limiters - Published Date: 19920101-; Population Group: Human  Search modes - SmartText Searching | 647 |
| S4 | Skin neoplasm* | Limiters - Date of Publication: 19920101-; Human  Search modes - Boolean/Phrase | 98,671 |
| S5 | (Skin adj2 neoplasm*) | Limiters - Published Date: 19920101-; Population Group: Human  Search modes - SmartText Searching | 173 |
| S6 | Skin (malignan* or skin tumo?r* or carcinoma*) | Limiters - Date of Publication: 19920101-; Human  Search modes - Boolean/Phrase | 12,279 |
| S7 | Cutaneous (malignan* or Skin tumo?r* or carcinoma*) | Limiters - Date of Publication: 19920101-; Human  Search modes - Boolean/Phrase | 13,469 |
| S8 | Melanoma* | Limiters - Date of Publication: 19920101-; Human  Search modes - Boolean/Phrase | 104,241 |
| S9 | Malignan* (melanoma* or m* or mm*) | Limiters - Date of Publication: 19920101-; Human  Search modes - Boolean/Phrase | 235,598 |
| S10 | Basal cell carcinoma* | Limiters - Date of Publication: 19920101-; Human  Search modes - Boolean/Phrase | 16,918 |
| S11 | (Basal cell adj2 carcinom*) | Limiters - Published Date: 19920101-; Population Group: Human  Search modes - SmartText Searching | 54 |
| S12 | Squamous cell carcinoma* | Limiters - Date of Publication: 19920101-; Human  Search modes - Boolean/Phrase | 133,586 |
| S13 | (Squamous cell adj2 carcinom*) | Limiters - Published Date: 19920101-; Population Group: Human  Search modes - SmartText Searching | 55 |
| S14 | (Sebaceous cell adj2 carcinoma*) | Limiters - Published Date: 19920101-; Population Group: Human  Search modes - SmartText Searching | 2,466 |
| S15 | Pigmented (N?ev* or lesion*) | Limiters - Date of Publication: 19920101-; Human  Search modes - Boolean/Phrase | 3,555 |
| S16 | Suspicious (lesion* or skin lesion* or mole* or n?ev*) | Limiters - Date of Publication: 19920101-; Human  Search modes - Boolean/Phrase | 4,766 |
| S17 | S1 OR S2 OR S3 OR S4 OR S5 OR S6 OR S7 OR S8 OR S9 OR S10 OR S11 OR S12 OR S13 OR S14 OR S15 OR S16 | Limiters - Date of Publication: 19920101-; Human  Search modes - Boolean/Phrase | 500,010 |
| S18 | (MH "Nurses") OR (MH "Nurse's Role") OR (MH "Practice Patterns, Nurses'") | Limiters - Date of Publication: 19920101-; Human  Search modes - Boolean/Phrase | 75,364 |
| S19 | Nurs* | Limiters - Date of Publication: 19920101-; Human  Search modes - Boolean/Phrase | 740,597 |
| S20 | S18 OR S19 | Limiters - Date of Publication: 19920101-; Human  Search modes - Boolean/Phrase | 740,597 |
| S21 | S17 AND S20 | Limiters - Date of Publication: 19920101-; Human  Search modes - Boolean/Phrase | 4,393 |
| S22 | (nurs* led) | Limiters - Date of Publication: 19920101-; Human  Search modes - Boolean/Phrase | 6,312 |
| S23 | S21 AND S22 | Limiters - Date of Publication: 19920101-; Human  Search modes - Boolean/Phrase | 50 |

### Search history from PubMed

This table shows the search performed on 8 May 2024

| **Search ID** | **Search Terms** | **Search Options** | **Results** |
| --- | --- | --- | --- |
| S1 | "Skin Neoplasms"[Mesh] | Filters Applied: Associated data, Humans, from 1992 - 3000/12/12 | 139,726 |
| S2 | Skin neoplasm* | Filters Applied: Associated data, Humans, from 1992 - 3000/12/12 | 138,754 |
| S3 | Skin cancer* | Filters Applied: Associated data, Humans, from 1992 - 3000/12/12 | 80,760 |
| S4 | Skin malignan* or skin tumour* or tumor* or carcinoma* | Filters Applied: Associated data, Humans, from 1992 - 3000/12/12 | 1,960,152 |
| S5 | Cutaneous (malignan* or skin tumour* or tumor* or carcinoma*) | Filters Applied: Associated data, Humans, from 1992 - 3000/12/12 | 37,916 |
| S6 | Melanoma* | Filters Applied: Associated data, Humans, from 1992 - 3000/12/12 | 105,515 |
| S7 | Malignan* (melanoma*) | Filters Applied: Associated data, Humans, from 1992 - 3000/12/12 | 31,943 |
| S8 | Basal cell carcinoma* | Filters Applied: Associated data, Humans, from 1992 - 3000/12/12 | 31,943 |
| S9 | Squamous cell carcinoma* | Filters Applied: Associated data, Humans, from 1992 - 3000/12/12 | 188,082 |
| S10 | Sebaceous cell carcinoma* | Filters Applied: Associated data, Humans, from 1992 - 3000/12/12 | 1,089 |
| S11 | Pigmented (nevi* or naevi or lesion*) | Filters Applied: Associated data, Humans, from 1992 - 3000/12/12 | 15,429 |
| S12 | Suspicious (lesion* or skin lesion* or mole* or nevi or naev*) | Filters Applied: Associated data, Humans, from 1992 - 3000/12/12 | 9,675 |
| S13 | "Skin Neoplasms"[Mesh] OR Skin neoplasm* OR Skin cancer* OR Skin malignan* or skin tumour* or tumor* or carcinoma* OR Cutaneous (malignan* or skin tumour* or tumor* or carcinoma*) OR Melanoma* OR Malignan* (melanoma*) OR Basal cell carcinoma OR Squamous cell carcinoma* OR Sebaceous cell carcinoma* OR Pigmented (nevi* or naevi or lesion*) OR Suspicious (lesion* or skin lesion* or mole* or nevi or naev*) | Filters Applied: Associated data, Humans, from 1992 - 3000/12/12 | 60,224 |
| S14 | Nurs* | Filters Applied: Associated data, Humans, from 1992 - 3000/12/12 | 746,200 |
| S15 | ("Skin Neoplasms"[Mesh] OR Skin neoplasm* OR Skin cancer* OR Skin malignan* or skin tumour* or tumor* or carcinoma* OR Cutaneous (malignan* or skin tumour* or tumor* or carcinoma*) OR Melanoma* OR Malignan* (melanoma*) OR Basal cell carcinoma OR Squamous cell carcinoma* OR Sebaceous cell carcinoma* OR Pigmented (nevi* or naevi or lesion*) OR Suspicious (lesion* or skin lesion* or mole* or nevi or naev*)) AND nurs* | Filters Applied: Associated data, Humans, from 1992 - 3000/12/12 | 311 |

### Search history from Embase

This table shows the search performed on 20 February 2024

| **Search ID** | **Search Terms** | **Search Options** | **Results** |
| --- | --- | --- | --- |
| S1 | skin tumor/ or non-melanoma skin cancer/ or skin cancer/ or squamous cell carcinoma/ or melanoma/ or Skin cancer*.mp. or basal cell carcinoma/ | limit to (human and yr="1992 -Current") | 292,471 |
| S2 | squamous cell carcinoma/ or melanoma/ or skin tumor/ or skin cancer/ or Skin neoplasm*.mp. or basal cell carcinoma/ | limit to (human and yr="1992 -Current") | 282,437 |
| S3 | cutaneous melanoma/ or melanoma/ or Melanoma*.mp. | limit to (human and yr="1992 -Current") | 198,462 |
| S4 | basal cell carcinoma/ or Basal cell carcinoma*.mp. | limit to (human and yr="1992 -Current") | 31,180 |
| S5 | squamous cell carcinoma/ or Squamous cell carcinoma*.mp. | limit to (human and yr="1992 -Current") | 209,736 |
| S6 | sebaceous carcinoma/ or Sebaceous cell carcinoma*.mp. | limit to (human and yr="1992 -Current") | 1,789 |
| S7 | S1 OR S2 OR S3 OR S4 OR S5 OR S6  skin tumor/ or non-melanoma skin cancer/ or skin cancer/ or squamous cell carcinoma/ or melanoma/ or Skin cancer*.mp. or basal cell carcinoma/ or squamous cell carcinoma/ or melanoma/ or skin tumor/ or skin cancer/ or Skin neoplasm*.mp. or basal cell carcinoma/ or cutaneous melanoma/ or melanoma/ or Melanoma*.mp. or basal cell carcinoma/ or Basal cell carcinoma*.mp. or squamous cell carcinoma/ or Squamous cell carcinoma*.mp. or sebaceous carcinoma/ or Sebaceous cell carcinoma*.mp. | limit to (human and yr="1992 -Current") | 542,158 |
| S8 | nursing education/ or nurse/ or Nurs*.mp. or nursing/ | limit to (human and yr="1992 -Current") | 679,600 |
| S9 | S7 AND S8  skin tumor/ or non-melanoma skin cancer/ or skin cancer/ or squamous cell carcinoma/ or melanoma/ or Skin cancer*.mp. or basal cell carcinoma/ or squamous cell carcinoma/ or melanoma/ or skin tumor/ or skin cancer/ or Skin neoplasm*.mp. or basal cell carcinoma/ or cutaneous melanoma/ or melanoma/ or Melanoma*.mp. or basal cell carcinoma/ or Basal cell carcinoma*.mp. or squamous cell carcinoma/ or Squamous cell carcinoma*.mp. or sebaceous carcinoma/ or Sebaceous cell carcinoma*.mp. AND nursing education/ or nurse/ or Nurs*.mp. or nursing/ | limit to (human and yr="1992 -Current") | 620 |
| S10 | nurs* led.mp. | limit to (human and yr="1992 -Current") | 8,719 |
| S11 | S9 AND S10  skin tumor/ or non-melanoma skin cancer/ or skin cancer/ or squamous cell carcinoma/ or melanoma/ or Skin cancer*.mp. or basal cell carcinoma/ or squamous cell carcinoma/ or melanoma/ or skin tumor/ or skin cancer/ or Skin neoplasm*.mp. or basal cell carcinoma/ or cutaneous melanoma/ or melanoma/ or Melanoma*.mp. or basal cell carcinoma/ or Basal cell carcinoma*.mp. or squamous cell carcinoma/ or Squamous cell carcinoma*.mp. or sebaceous carcinoma/ or Sebaceous cell carcinoma*.mp. AND nursing education/ or nurse/ or Nurs*.mp. or nursing/ AND nurs* led.mp. | limit to (human and yr="1992 -Current") | 0 |

### Search history from CINAHL Ultimate

This table shows the search performed on 21 February 2024

| **Search ID** | **Search Terms** | **Search Options** | **Results** |
| --- | --- | --- | --- |
| S1 | (MH "Skin Neoplasms") OR (MH "Neoplasms, Adnexal and Skin Appendage") | Limiters - Date of Publication: 19920101-; Human  Search modes - Boolean/Phrase | 5,327 |
| S2 | Skin cancer* | Limiters - Date of Publication: 19920101-; Human  Search modes - Boolean/Phrase | 6,005 |
| S3 | (Skin adj2 cancer*) | Limiters - Published Date: 19920101-; Population Group: Human  Search modes - SmartText Searching | 83 |
| S4 | Skin neoplasm* | Limiters - Date of Publication: 19920101-; Human  Search modes - Boolean/Phrase | 5,368 |
| S5 | (Skin adj2 neoplasm*) | Limiters - Published Date: 19920101-; Population Group: Human  Search modes - SmartText Searching | 23 |
| S6 | Skin (malignan* or skin tumo?r* or carcinoma*) | Limiters - Date of Publication: 19920101-; Human  Search modes - Boolean/Phrase | 737 |
| S7 | Cutaneous (malignan* or Skin tumo?r* or carcinoma*) | Limiters - Date of Publication: 19920101-; Human  Search modes - Boolean/Phrase | 710 |
| S8 | Melanoma* | Limiters - Date of Publication: 19920101-; Human  Search modes - Boolean/Phrase | 7,060 |
| S9 | Malignan* (melanoma* or m* or mm*) | Limiters - Date of Publication: 19920101-; Human  Search modes - Boolean/Phrase | 18,669 |
| S10 | Basal cell carcinoma* | Limiters - Date of Publication: 19920101-; Human  Search modes - Boolean/Phrase | 991 |
| S11 | (Basal cell adj2 carcinom*) | Limiters - Published Date: 19920101-; Population Group: Human  Search modes - SmartText Searching | 1 |
| S12 | Squamous cell carcinoma* | Limiters - Date of Publication: 19920101-; Human  Search modes - Boolean/Phrase | 14,851 |
| S13 | (Squamous cell adj2 carcinom*) | Limiters - Published Date: 19920101-; Population Group: Human  Search modes - SmartText Searching | 1 |
| S14 | (Sebaceous cell adj2 carcinoma*) | Limiters - Published Date: 19920101-; Population Group: Human  Search modes - SmartText Searching | 72 |
| S15 | Pigmented (N?ev* or lesion*) | Limiters - Date of Publication: 19920101-; Human  Search modes - Boolean/Phrase | 181 |
| S16 | Suspicious (lesion* or skin lesion* or mole* or n?ev*) | Limiters - Date of Publication: 19920101-; Human  Search modes - Boolean/Phrase80 | 620 |
| S17 | S1 OR S2 OR S3 OR S4 OR S5 OR S6 OR S7 OR S8 OR S9 OR S10 OR S11 OR S12 OR S13 OR S14 OR S15 OR S16 | Limiters - Date of Publication: 19920101-Human  Search modes - Boolean/Phrase | 26,098 |
| S18 | (MH "Nurses") OR (MH "Nurse's Role") OR (MH "Practice Patterns, Nurses'") | Limiters - Date of Publication: 19920101-; Human  Search modes - Boolean/Phrase | 17,671 |
| S19 | Nurs* | Limiters - Date of Publication: 19920101-; Human  Search modes - Boolean/Phrase | 258,636 |
| S20 | S18 OR S19 | Limiters - Date of Publication: 19920101-; Human  Search modes - Boolean/Phrase | 258,636 |
| S21 | S17 AND S20 | Limiters - Date of Publication: 19920101-; Human  Search modes - Boolean/Phrase | 418 |
| S22 | (nurs* led) | Limiters - Date of Publication: 19920101-; Human  Search modes - Boolean/Phrase | 4,301 |
| S23 | S21 AND S22 | Limiters - Date of Publication: 19920101-; Human  Search modes - Boolean/Phrase | 12 |

### Search history from Science Direct

This table shows the search performed on 21 February 2023

| **Search ID** | **Search Terms** | **Search Options** | **Results** |
| --- | --- | --- | --- |
| S1 | skin cancer | Limit: 1992-2024 | 417,150 |
| S2 | skin neoplasm | Limit: 1992-2024 | 72,742 |
| S3 | skin AND tumor OR tumour OR carcinoma OR malignancy | Limit: 1992-2024 | 1,000,000+ |
| S4 | cutaneous malignancy or Skin tumour or tumor or carcinoma | Limit: 1992-2024 | 20,998 |
| S5 | Melanoma | Limit: 1992-2024 | 214,789 |
| S6 | malignant AND melanoma | Limit: 1992-2024 | 99,608 |
| S7 | basal cell carcinoma | Limit: 1992-2024 | 491,752 |
| S8 | Squamous cell carcinoma | Limit: 1992-2024 | 199,836 |
| S9 | sebaceous cell carcinoma | Limit: 1992-2024 | 8,891 |
| S10 | pigmented AND nevi OR naevus OR lesion | Limit: 1992-2024 | 1,000,000+ |
| S11 | suspicious lesion or skin lesion or mole or nevi or naevus | Limit: 1992-2024 | 22 |
| S12 | skin cancer or skin neoplasm or melanoma or basal cell carcinoma or squamous cell carcinoma or sebaceous cell carcinoma or pigmented lesion | Limit: 1992-2024 | 943 |
| S13 | Nurse led | Limit: 1992-2024 | 121,361 |
| S14 | skin cancer or skin neoplasm or melanoma or basal cell carcinoma or squamous cell carcinoma or sebaceous cell carcinoma or pigmented lesion AND nurse | Limit: 1992-2024 | 63 |

### Search history from Scopus

This table shows the search performed on 21 February 2024

| **Search ID** | **Search Terms** | **Search Options** | **Results** |
| --- | --- | --- | --- |
| S1 | Skin AND cancer | TITLE-ABS-KEY ( skin  AND cancer )  AND  PUBYEAR  >  1991  AND  PUBYEAR  <  2025  AND  ( LIMIT-TO ( EXACTKEYWORD ,  "Human" ) ) | 162,857 |
| S2 | Skin AND neoplasm | TITLE-ABS-KEY ( skin  AND neoplasm )  AND  PUBYEAR  >  1991  AND  PUBYEAR  <  2025  AND  ( LIMIT-TO ( EXACTKEYWORD ,  "Human" ) ) | 151,461 |
| S3 | cutaneous AND malignancy OR skin AND tumour OR skin AND tumor OR skin AND carcinoma OR cutaneous AND carcinoma OR skin AND malignancy | TITLE-ABS-KEY ( cutaneous  AND malignancy  OR  skin  AND tumour  OR  skin  AND tumor  OR  skin  AND carcinoma  OR  cutaneous  AND carcinoma  OR  skin  AND malignancy )  AND  PUBYEAR  >  1991  AND  PUBYEAR  <  2025  AND  ( LIMIT-TO ( EXACTKEYWORD ,  "Human" ) ) | 7,074 |
| S4 | Melanoma | TITLE-ABS-KEY ( melanoma ) AND PUBYEAR > 1991 AND PUBYEAR < 2025 AND ( LIMIT-TO ( EXACTKEYWORD , "Human" ) ) | 157,006 |
| S5 | Malignant AND melanoma | TITLE-ABS-KEY ( malignant AND melanoma ) AND PUBYEAR > 1991 AND PUBYEAR < 2025 AND ( LIMIT-TO ( EXACTKEYWORD , "Human" ) ) | 41,817 |
| S6 | basal AND cell AND carcinoma | TITLE-ABS-KEY ( basal AND cell AND carcinoma ) AND PUBYEAR > 1991 AND PUBYEAR < 2025 AND ( LIMIT-TO ( EXACTKEYWORD , "Human" ) ) | 35,614 |
| S7 | squamous AND cell AND carcinoma | TITLE-ABS-KEY ( squamous  AND cell  AND carcinoma )  AND  PUBYEAR  >  1991  AND  PUBYEAR  <  2025  AND  ( LIMIT-TO ( EXACTKEYWORD ,  "Human" ) ) | 195,679 |
| S8 | sebaceous AND cell AND carcinoma | TITLE-ABS-KEY ( sebaceous AND cell AND carcinoma ) AND PUBYEAR > 1991 AND PUBYEAR < 2025 AND ( LIMIT-TO ( EXACTKEYWORD , "Human" ) ) | 2,128 |
| S9 | pigmented AND nevi OR pigmented AND naevus OR pigmented AND lesion OR pigmented AND skin AND lesion | TITLE-ABS-KEY ( pigmented AND nevi OR pigmented AND naevus OR pigmented AND lesion OR pigmented AND skin AND lesion ) AND PUBYEAR > 1991 AND PUBYEAR < 2025 AND ( LIMIT-TO ( EXACTKEYWORD , "Human" ) ) | 5700 |
| S10 | suspicious AND lesion OR suspicious AND skin AND lesion OR suspicious AND mole OR suspicious AND nevi OR suspicious AND naevus | TITLE-ABS KEY ( suspicious  AND lesion  OR  suspicious  AND skin  AND lesion  OR  suspicious  AND mole  OR  suspicious  AND nevi  OR  suspicious  AND naevus )  AND  PUBYEAR  >  1991  AND  PUBYEAR  <  2025  AND  ( LIMIT-TO ( EXACTKEYWORD ,  "Human" ) ) | 326 |
| S11 | skin AND cancer OR suspicious AND lesion OR suspicious AND skin AND lesion OR suspicious AND mole OR suspicious AND nevi OR suspicious AND naevus pigmented AND nevi OR pigmented AND naevus OR pigmented AND lesion OR pigmented AND skin AND lesion OR sebaceous OR squamous OR basal AND cell AND carcinoma OR malignant AND melanoma OR melanoma OR cutaneous AND malignancy OR skin AND tumour OR skin AND tumor OR skin AND carcinoma OR cutaneous AND carcinoma OR skin AND malignancy OR skin AND neoplasm | ( TITLE-ABS-KEY ( skin AND cancer ) OR TITLE-ABS-KEY ( suspicious AND lesion OR suspicious AND skin AND lesion OR suspicious AND mole OR suspicious AND nevi OR suspicious AND naevus ) OR TITLE-ABS-KEY ( pigmented AND nevi OR pigmented AND naevus OR pigmented AND lesion OR pigmented AND skin AND lesion ) OR TITLE-ABS-KEY ( sebaceous OR squamous OR basal AND cell AND carcinoma ) OR TITLE-ABS-KEY ( malignant AND melanoma ) OR TITLE-ABS-KEY ( melanoma ) OR TITLE-ABS-KEY ( cutaneous AND malignancy OR skin AND tumour OR skin AND tumor OR skin AND carcinoma OR cutaneous AND carcinoma OR skin AND malignancy ) OR TITLE-ABS-KEY ( skin AND neoplasm ) ) AND PUBYEAR > 1991 AND PUBYEAR < 2024 | 571,885 |
| S12 | Nurs* | TITLE-ABS-KEY ( nurs* ) AND PUBYEAR > 1991 AND PUBYEAR < 2025 AND ( LIMIT-TO ( EXACTKEYWORD , "Human" ) ) | 576,625 |
| S13 | nurs* AND led | TITLE-ABS-KEY ( nurs* AND led ) AND PUBYEAR > 1991 AND PUBYEAR < 2025 AND ( LIMIT-TO ( EXACTKEYWORD , "Human" ) ) | 15,542 |
| S14 | skin AND cancer OR suspicious AND lesion OR suspicious AND skin AND lesion OR suspicious AND mole OR suspicious AND nevi OR suspicious AND naevus OR pigmented AND nevi OR pigmented AND naevus OR pigmented AND lesion OR pigmented AND skin AND lesion OR sebaceous OR squamous OR basal AND cell AND carcinoma OR malignant AND melanoma OR melanoma OR cutaneous AND malignancy OR skin AND tumour OR skin AND tumor OR skin AND carcinoma OR cutaneous AND carcinoma OR skin AND malignancy OR skin AND neoplasm AND nurs* | ( TITLE-ABS-KEY ( skin AND cancer ) OR TITLE-ABS-KEY ( suspicious AND lesion OR suspicious AND skin AND lesion OR suspicious AND mole OR suspicious AND nevi OR suspicious AND naevus ) OR TITLE-ABS-KEY ( pigmented AND nevi OR pigmented AND naevus OR pigmented AND lesion OR pigmented AND skin AND lesion ) OR TITLE-ABS-KEY ( sebaceous OR squamous OR basal AND cell AND carcinoma ) OR TITLE-ABS-KEY ( malignant AND melanoma ) OR TITLE-ABS-KEY ( melanoma ) OR TITLE-ABS-KEY ( cutaneous AND malignancy OR skin AND tumour OR skin AND tumor OR skin AND carcinoma OR cutaneous AND carcinoma OR skin AND malignancy ) OR TITLE-ABS-KEY ( skin AND neoplasm ) AND TITLE-ABS-KEY ( nurs* ) ) AND PUBYEAR > 1991 AND PUBYEAR < 2024 | 2,785 |
| S15 | skin AND cancer OR suspicious AND lesion OR suspicious AND skin AND lesion OR suspicious AND mole OR suspicious AND nevi OR suspicious AND naevus OR pigmented AND nevi OR pigmented AND naevus OR pigmented AND lesion OR pigmented AND skin AND lesion OR sebaceous OR squamous OR basal AND cell AND carcinoma OR malignant AND melanoma OR melanoma OR cutaneous AND malignancy OR skin AND tumour OR skin AND tumor OR skin AND carcinoma OR cutaneous AND carcinoma OR skin AND malignancy OR skin AND neoplasm AND nurs* AND led | ( TITLE-ABS-KEY ( skin AND cancer ) OR TITLE-ABS-KEY ( suspicious AND lesion OR suspicious AND skin AND lesion OR suspicious AND mole OR suspicious AND nevi OR suspicious AND naevus ) OR TITLE-ABS-KEY ( pigmented AND nevi OR pigmented AND naevus OR pigmented AND lesion OR pigmented AND skin AND lesion ) OR TITLE-ABS-KEY ( sebaceous OR squamous OR basal AND cell AND carcinoma ) OR TITLE-ABS-KEY ( malignant AND melanoma ) OR TITLE-ABS-KEY ( melanoma ) OR TITLE-ABS-KEY ( cutaneous AND malignancy OR skin AND tumour OR skin AND tumor OR skin AND carcinoma OR cutaneous AND carcinoma OR skin AND malignancy ) OR TITLE-ABS-KEY ( skin AND neoplasm ) AND TITLE-ABS-KEY ( nurs* AND led ) ) AND PUBYEAR > 1991 AND PUBYEAR < 2024 | 84 |

### Search history from British Nursing Index

This table shows the search performed on 21 February 2024

| **Search ID** | **Search Terms** | **Search Options** | **Results** |
| --- | --- | --- | --- |
| S1 | Skin cancer* | Narrowed by: Entered date: 1992-01-01 – 2024 | 13,435 |
| S2 | Skin neoplasm* | Narrowed by: Entered date: 1992-01-01 – 2024 | 2,256 |
| S3 | Skin (malignan* or skin tumo?r* or carcinoma*) | Narrowed by: Entered date: 1992-01-01 – 2024 | 9,684 |
| S4 | Cutaneous (malignan* or Skin tumo?r* or carcinoma*) | Narrowed by: Entered date: 1992-01-01 – 2024 | 2,278 |
| S5 | Melanoma* | Narrowed by: Entered date: 1992-01-01 – 2024 | 4,068 |
| S6 | Malignan* melanoma* | Narrowed by: Entered date: 1992-01-01 – 2024 | 2,222 |
| S7 | Basal cell carcinoma* | Narrowed by: Entered date: 1992-01-01 – 2024 | 1,275 |
| S8 | Squamous cell carcinoma* | Narrowed by: Entered date: 1992-01-01 – 2024 | 2,324 |
| S9 | Sebaceous cell carcinoma* | Narrowed by: Entered date: 1992-01-01 – 2024 | 111 |
| S10 | Pigmented (N?ev* or lesion*) | Narrowed by: Entered date: 1992-01-01 – 2024 | 627 |
| S11 | Suspicious (lesion* or skin lesion* or mole* or n?ev*) | Narrowed by: Entered date: 1992-01-01 – 2024 | 1,948 |
| S12 | (Skin cancer*) OR (Skin neoplasm*) OR (Skin (malignan* OR skin tumo?r* OR carcinoma*)) OR (Cutaneous (malignan* OR Skin tumo?r* OR carcinoma*)) OR Melanoma* OR (Malignan* melanoma*) OR (Basal cell carcinoma*) OR (Squamous cell carcinoma*) OR (Sebaceous cell carcinoma*) OR (Pigmented (N?ev* OR lesion*)) OR (Suspicious (lesion* OR skin lesion* OR mole* OR n?ev*)) | Narrowed by: Entered date: 1992-01-01 - 2024 | 16,946 |
| S13 | Nurs* | Narrowed by: Entered date: 1992-01-01 – 2024 | 742,238 |
| S14 | (Skin cancer*) OR (Skin neoplasm*) OR (Skin (malignan* OR skin tumo?r* OR carcinoma*)) OR (Cutaneous (malignan* OR Skin tumo?r* OR carcinoma*)) OR Melanoma* OR (Malignan* melanoma*) OR (Basal cell carcinoma*) OR (Squamous cell carcinoma*) OR (Sebaceous cell carcinoma*) OR (Pigmented (N?ev* OR lesion*)) OR (Suspicious (lesion* OR skin lesion* OR mole* OR n?ev*)) AND nurs* | Narrowed by: Entered date: 1992-01-01 – 2024 | 11,717 |
| S15 | (Nurs* led) | Narrowed by: Entered date: 1992-01-01 – 2024 | 63,680 |
| S16 | (Skin cancer*) OR (Skin neoplasm*) OR (Skin (malignan* OR skin tumo?r* OR carcinoma*)) OR (Cutaneous (malignan* OR Skin tumo?r* OR carcinoma*)) OR Melanoma* OR (Malignan* melanoma*) OR (Basal cell carcinoma*) OR (Squamous cell carcinoma*) OR (Sebaceous cell carcinoma*) OR (Pigmented (N?ev* OR lesion*)) OR (Suspicious (lesion* OR skin lesion* OR mole* OR n?ev*)) AND (Nurs* led) | Narrowed by: Entered date: 1992-01-01 – 2024 | 2,902 |
| S17 | (Skin cancer*) OR (Skin neoplasm*) OR (Skin (malignan* OR skin tumo?r* OR carcinoma*)) OR (Cutaneous (malignan* OR Skin tumo?r* OR carcinoma*)) OR Melanoma* OR (Malignan* melanoma*) OR (Basal cell carcinoma*) OR (Squamous cell carcinoma*) OR (Sebaceous cell carcinoma*) OR (Pigmented (N?ev* OR lesion*)) OR (Suspicious (lesion* OR skin lesion* OR mole* OR n?ev*)) AND (Nurs* led) AND (skin cancer) | Narrowed by: Entered date: 1992-01-01 - 2024 | 2036 |
| S18 | (Skin cancer*) OR (Skin neoplasm*) OR (Skin (malignan* OR skin tumo?r* OR carcinoma*)) OR (Cutaneous (malignan* OR Skin tumo?r* OR carcinoma*)) OR Melanoma* OR (Malignan* melanoma*) OR (Basal cell carcinoma*) OR (Squamous cell carcinoma*) OR (Sebaceous cell carcinoma*) OR (Pigmented (N?ev* OR lesion*)) OR (Suspicious (lesion* OR skin lesion* OR mole* OR n?ev*)) AND (Nurs* led) AND (skin cancer) NOT oncology | Narrowed by: Entered date: 1992-01-01 – 2024 | 1120 |

### Search history from Latin American and Caribbean Health Sciences Literature (LILACS)

This table shows the search performed on 21 February 2024

| **Search ID** | **Search Terms** | **Search Options** | **Results** |
| --- | --- | --- | --- |
| S1 | Skin cancer* | skin cancer* AND ( db:("LILACS")) AND (year_cluster:[1992 TO 2024]) | 3,812 |
| S2 | “Skin cancer” | “skin cancer” AND ( db:("LILACS")) AND (year_cluster:[1992 TO 2024]) | 3,747 |
| S3 | Skin neoplasm* | skin neoplasm* AND ( db:("LILACS")) AND (year_cluster:[1992 TO 2024]) | 3,715 |
| S4 | “Skin neoplasm” | “skin neoplasm” AND ( db:("LILACS")) AND (year_cluster:[1992 TO 2024]) | 3,356 |
| S5 | Skin (malignan* or skin tumo?r* or carcinoma*) | skin (malignan* OR skin tumo?r* OR carcinoma*) AND ( db:("LILACS")) AND (year_cluster:[1992 TO 2024]) | 20,844 |
| S6 | Cutaneous (malignan* or Skin tumo?r* or carcinoma*) | cutaneous (malignan* OR skin tumo?r* OR carcinoma*) AND ( db:("LILACS")) AND (year_cluster:[1992 TO 2024]) | 4,214 |
| S7 | Melanoma* | melanoma* AND ( db:("LILACS")) AND (year_cluster:[1992 TO 2024]) | 2,380 |
| S8 | Malignan* melanoma | malignan* melanoma AND ( db:("LILACS")) AND (year_cluster:[1992 TO 2024]) | 1,704 |
| S9 | Basal cell carcinoma* | basal cell carcinoma* AND ( db:("LILACS")) AND (year_cluster:[1992 TO 2024]) | 3,299 |
| S10 | “Basal cell carcinoma” | “basal cell carcinoma” AND ( db:("LILACS")) AND (year_cluster:[1992 TO 2024]) | 3,280 |
| S11 | Squamous cell carcinom* | squamous cell carcinom* AND ( db:("LILACS")) AND (year_cluster:[1992 TO 2024]) | 3,437 |
| S12 | “Squamous cell carcinoma” | “squamous cell carcinoma” AND ( db:("LILACS")) AND (year_cluster:[1992 TO 2024]) | 3,385 |
| S13 | Sebaceous cell carcinoma* | sebaceous cell carcinoma* AND ( db:("LILACS")) AND (year_cluster:[1992 TO 2024]) | 136 |
| S14 | “Sebaceous cell carcinoma” | “sebaceous cell carcinoma” AND ( db:("LILACS")) AND (year_cluster:[1992 TO 2024]) | 135 |
| S15 | Pigmented (N?ev* or lesion*) | pigmented (n?ev* OR lesion*) AND ( db:("LILACS")) AND (year_cluster:[1992 TO 2024]) | 770 |
| S16 | Suspicious (lesion* or skin lesion* or mole* or n?ev*) | suspicious (lesion* OR skin lesion* OR mole* OR n?ev*) AND ( db:("LILACS")) AND (year_cluster:[1992 TO 2024]) | 354 |
| S17 | Skin cancer* OR “Skin cancer” OR Skin neoplasm* OR “Skin neoplasm” OR Skin (malignan* or skin tumo?r* or carcinoma*) OR Cutaneous (malignan* or Skin tumo?r* or carcinoma*) OR Melanoma* OR Malignan* melanoma OR Basal cell carcinoma* OR “Basal cell carcinoma” OR Squamous cell carcinom* OR “Squamous cell carcinoma” OR Sebaceous cell carcinoma* OR “Sebaceous cell carcinoma” OR Pigmented (N?ev* or lesion*) OR Suspicious (lesion* or skin lesion* or mole* or n?ev*) | skin cancer* OR “skin cancer” OR skin neoplasm* OR “skin neoplasm” OR skin (malignan* OR skin tumo?r* OR carcinoma*) OR cutaneous (malignan* OR skin tumo?r* OR carcinoma*) OR melanoma* OR malignan* melanoma OR basal cell carcinoma* OR “basal cell carcinoma” OR squamous cell carcinom* OR “squamous cell carcinoma” OR sebaceous cell carcinoma* OR “sebaceous cell carcinoma” OR pigmented (n?ev* OR lesion*) OR suspicious (lesion* OR skin lesion* OR mole* OR n?ev*) AND ( db:("LILACS")) AND (year_cluster:[1992 TO 2024]) | 3,245 |
| S18 | nurs* | nurs* AND ( db:("LILACS")) AND (year_cluster:[1992 TO 2024]) | 63,278 |
| S19 | nurs* led | nurs* led AND ( db:("LILACS")) AND (year_cluster:[1992 TO 2024]) | 809 |
| S20 | (Skin cancer* OR “Skin cancer” OR Skin neoplasm* OR “Skin neoplasm” OR Skin (malignan* or skin tumo?r* or carcinoma*) OR Cutaneous (malignan* or Skin tumo?r* or carcinoma*) OR Melanoma* OR Malignan* melanoma OR Basal cell carcinoma* OR “Basal cell carcinoma” OR Squamous cell carcinom* OR “Squamous cell carcinoma” OR Sebaceous cell carcinoma* OR “Sebaceous cell carcinoma” OR Pigmented (N?ev* or lesion*) OR Suspicious (lesion* or skin lesion* or mole* or n?ev*)) AND (nurs*) | ( (skin cancer* OR “skin cancer” OR skin neoplasm* OR “skin neoplasm” OR skin (malignan* OR skin tumo?r* OR carcinoma*) OR cutaneous (malignan* OR skin tumo?r* OR carcinoma*) OR melanoma* OR malignan* melanoma OR basal cell carcinoma* OR “basal cell carcinoma” OR squamous cell carcinom* OR “squamous cell carcinoma” OR sebaceous cell carcinoma* OR “sebaceous cell carcinoma” OR pigmented (n?ev* OR lesion*) OR suspicious (lesion* OR skin lesion* OR mole* OR n?ev*)) ) AND ((nurs*) ) AND (year_cluster:[1992 TO 2024]) | 698 |
| S21 | (Skin cancer* OR “Skin cancer” OR Skin neoplasm* OR “Skin neoplasm” OR Skin (malignan* or skin tumo?r* or carcinoma*) OR Cutaneous (malignan* or Skin tumo?r* or carcinoma*) OR Melanoma* OR Malignan* melanoma OR Basal cell carcinoma* OR “Basal cell carcinoma” OR Squamous cell carcinom* OR “Squamous cell carcinoma” OR Sebaceous cell carcinoma* OR “Sebaceous cell carcinoma” OR Pigmented (N?ev* or lesion*) OR Suspicious (lesion* or skin lesion* or mole* or n?ev*)) AND (nurs* led) | ( (skin cancer* OR “skin cancer” OR skin neoplasm* OR “skin neoplasm” OR skin (malignan* OR skin tumo?r* OR carcinoma*) OR cutaneous (malignan* OR skin tumo?r* OR carcinoma*) OR melanoma* OR malignan* melanoma OR basal cell carcinoma* OR “basal cell carcinoma” OR squamous cell carcinom* OR “squamous cell carcinoma” OR sebaceous cell carcinoma* OR “sebaceous cell carcinoma” OR pigmented (n?ev* OR lesion*) OR suspicious (lesion* OR skin lesion* OR mole* OR n?ev*)) ) AND ((nurs* led) ) AND (year_cluster:[1992 TO 2024]) | 17 |

### Search history from ****APA PsycInfo****

This table shows the search performed **on 23 February 2024**

| **Search ID** | **Search Terms** | **Search Options** | **Results** |
| --- | --- | --- | --- |
| S1 | Skin cancer* | Applied filters: 1992-01-01 - 2024-02-21 | 1984 |
| S2 | Skin neoplasm* | Applied filters: 1992-01-01 - 2024-02-21 | 1,534 |
| S3 | Melanoma* | Applied filters: 1992-01-01 - 2024-02-21 | 1,226 |
| S4 | Basal cell carcinoma* | Applied filters: 1992-01-01 - 2024-02-21 | 93 |
| S5 | Squamous cell carcinoma* | Applied filters: 1992-01-01 - 2024-02-21 | 358 |
| S6 | Sebaceous cell carcinoma* | Applied filters: 1992-01-01 - 2024-02-21 | 0 |
| S7 | Pigmented (N?ev* or lesion*) | Applied filters: 1992-01-01 - 2024-02-21 | 79 |
| S8 | Suspicious (lesion* or skin lesion* or mole* or n?ev*) | Applied filters: 1992-01-01 - 2024-02-21 | 119 |
| S9 | S1 OR S2 OR S3 OR S4 OR S5 OR S6 OR S7 OR S8  Skin cancer* OR Skin neoplasm* OR Melanoma* OR Basal cell carcinoma* OR Squamous cell carcinoma* OR Sebaceous cell carcinoma* OR Pigmented (N?ev* or lesion*) OR Suspicious (lesion* or skin lesion* or mole* or n?ev*) | Applied filters: 1992-01-01 - 2024-02-21 | 24,629 |
| S10 | (MH "Nurses") OR (MH "Nurse's Role") OR (MH "Practice Patterns, Nurses'") | Applied filters: 1992-01-01 - 2024-02-21 | 71 |
| S11 | Nurs* | Applied filters: 1992-01-01 - 2024-02-21 | 190,762 |
| S12 | Nurs* led | Applied filters: 1992-01-01 - 2024-02-21 | 5657 |
|  | (skin cancer*) OR (Skin neoplasm*) OR Melanoma* OR (Basal cell carcinoma*) OR (Squamous cell carcinoma*) OR (Sebaceous cell carcinoma*) OR (Pigmented (N?ev* OR lesion*)) OR (Suspicious (lesion* OR skin lesion* OR mole* OR n?ev*)) AND (nurs* led) | Applied filters: 1992-01-01 - 2024-02-21 | 3228 |
| S13 | (Suspicious (lesion* OR skin lesion* OR mole* OR n?ev*)) OR (Skin neoplasm*) OR Melanoma* OR (Basal cell carcinoma*) OR (Squamous cell carcinoma*) OR (Sebaceous cell carcinoma*) OR (Pigmented (N?ev* OR lesion*)) AND (skin cancer*) AND (nurs* led) NOT oncology | Applied filters: 1992-01-01 - 2024-02-21 | 2280 |

### Search history from ****ERIC**** the Education Resource Information Centre

This table shows the search performed on **23 February 2024**

| **Search ID** | **Search Terms** | **Results** |
| --- | --- | --- |
| S1 | Skin cancer | 114 |
| S2 | Skin neoplasm | 0 |
| S3 | Melanoma | 32 |
| S4 | Basal cell carcinoma | 2 |
| S5 | Squamous cell carcinoma | 1 |
| S6 | Sebaceous cell carcinoma | 0 |
| S7 | Pigmented lesion | 0 |
| S9 | Suspicious lesion | 0 |
| S10 | Skin cancer OR Melanoma OR Basal cell carcinoma OR Squamous cell carcinoma | 10,589 |
| S11 | Nurse | 9,073 |
| S12 | Nurse led | 130 |
| S13 | Skin cancer AND nurse | 90 |

### Search history from Trip Medical Database

This table shows the search performed on 22 February 2023

| **Search ID** | **Search Terms** | **Search Options** | **Results** |
| --- | --- | --- | --- |
| S1 | Skin cancer | date:1992 to_date:2024 | 70,133 |
| S2 | Skin neoplasm | date:1992 to_date:2024 | 27,831 |
| S3 | Melanoma | date:1992 to_date:2024 | 39,527 |
| S4 | Squamous cell carcinoma | date:1992 to_date:2024 | 43,035 |
| S5 | Basal cell carcinoma | date:1992 to_date:2024 | 19,434 |
| S6 | Sebaceous cell carcinoma | date:1992 to_date:2024 | 918 |
| S7 | Skin cancer OR Skin neoplasm OR Melanoma OR Squamous cell carcinoma OR Basal cell carcinoma OR Sebaceous cell carcinoma | date:1992 to_date:2024 | 76,549 |
| S8 | Nurse | date:1992 to_date:2024 | 133,499 |
| S9 | Nurse led | date:1992 to_date:2024 | 13,302 |
| S10 | Skin cancer OR Skin neoplasm OR Melanoma OR Squamous cell carcinoma OR Basal cell carcinoma OR Sebaceous cell carcinoma AND nurse | date:1992 to_date:2024 | 10,082 |
| S11 | Skin cancer OR Skin neoplasm OR Melanoma OR Squamous cell carcinoma OR Basal cell carcinoma OR Sebaceous cell carcinoma AND nurse led | date:1992 to_date:2024 | 184 |

### EThOs (E-Theses Online Service)

This table shows the search performed on 11 July 2023 *(unable to re-run search due to cyber-attack)*

| **Search ID** | **Search Terms** | **Search Options** | **Results** |
| --- | --- | --- | --- |
| S1  (Single search) | skin cancer OR skin neoplasm OR melanoma OR squamous cell carcinoma OR sebaceous cell carcinoma OR basal cell carcinoma | Words  Limits 1992- 11/07/2023 | 1094 |

### Search history from Web of Science (from Web of Science Core Collection)

This table shows the search performed on 29 February 2024

| **Search ID** | **Search Terms** | **Search Options** | **Results** |
| --- | --- | --- | --- |
| S1 | Skin cancer* | Timespan: 1992-01-01 to 2024-02-29 (Publication Date) | 146,784 |
| S2 | Skin neoplasm* | Timespan: 1992-01-01 to 2024-02-29 (Publication Date) | 209,603 |
| S3 | Melanoma* | Timespan: 1992-01-01 to 2024-02-29 (Publication Date) | 240,716 |
| S4 | Malignan* melanoma* | Timespan: 1992-01-01 to 2024-02-29 (Publication Date) | 78,422 |
| S5 | Squamous cell carcinoma* | Timespan: 1992-01-01 to 2024-02-29 (Publication Date) | 226,933 |
| S6 | Basal cell carcinoma* | Timespan: 1992-01-01 to 2024-02-29 (Publication Date) | 40,793 |
| S7 | Sebaceous cell carcinoma* | Timespan: 1992-01-01 to 2024-02-29 (Publication Date) | 2,017 |
| S8 | Skin (malignan* or skin tumo?r* or carcinoma*) | Timespan: 1992-01-01 to 2024-02-29 (Publication Date) | 135,503 |
| S9 | Cutaneous (malignan* or Skin tumo?r* or carcinoma*) | Timespan: 1992-01-01 to 2024-02-29 (Publication Date) | 50,717 |
| S10 | Pigmented (nevi or naevi or nevus or naevus or lesion*) | Timespan: 1992-01-01 to 2024-02-29 (Publication Date) | 22,596 |
| S11 | Suspicious (lesion* or skin lesion* or cutaneous lesion* mole* or nevi or naevi or nevus or naevus) | Timespan: 1992-01-01 to 2024-02-29 (Publication Date) | 12,226 |
| S13 | Skin cancer* OR Skin neoplasm* OR Melanoma* OR Malignan* melanoma* OR Squamous cell carcinoma* OR Basal cell carcinoma* OR Sebaceous cell carcinoma* OR Skin (malignan* or skin tumo?r* or carcinoma*) OR Cutaneous (malignan* or Skin tumo?r* or carcinoma*) OR Pigmented (nevi or naevi or nevus or nevus or lesion*) OR Suspicious (lesion* or skin lesion* or cutaneous lesion* mole* or nevi or naevi or nevus or nevus) | Timespan: 1992-01-01 to 2024-02-29 (Publication Date) | 676,716 |
| S15 | Nurs* | Timespan: 1992-01-01 to 2024-02-29 (Publication Date) | 642,607 |
| S16 | Nurs* led | Timespan: 1992-01-01 to 2024-02-29 (Publication Date) | 64,497 |
|  | Skin cancer* OR Skin neoplasm* OR Melanoma* OR Malignan* melanoma* OR Squamous cell carcinoma* OR Basal cell carcinoma* OR Sebaceous cell carcinoma* OR Skin (malignan* or skin tumo?r* or carcinoma*) OR Cutaneous (malignan* or Skin tumo?r* or carcinoma*) OR Pigmented (nevi or naevi or nevus or naevus or lesion*) OR Suspicious (lesion* or skin lesion* or cutaneous lesion* mole* or nevi or naevi or nevus or naevus) AND nurs* | Timespan: 1992-01-01 to 2024-02-29 (Publication Date) | 2,514 |
| S17 | Skin cancer* OR Skin neoplasm* OR Melanoma* OR Malignan* melanoma* OR Squamous cell carcinoma* OR Basal cell carcinoma* OR Sebaceous cell carcinoma* OR Skin (malignan* or skin tumo?r* or carcinoma*) OR Cutaneous (malignan* or Skin tumo?r* or carcinoma*) OR Pigmented (nevi or naevi or nevus or naevus or lesion*) OR Suspicious (lesion* or skin lesion* or cutaneous lesion* mole* or nevi or naevi or nevus or naevus) AND nurs* led | Timespan: 1992-01-01 to 2024-02-29 (Publication Date) | 352 |

### [Search history from Google](https://www.cochranelibrary.com/) Scholar

This table shows the search performed on 22 February 2023

| **Search ID** | **Search Terms** | **Search Options** | **Results** |
| --- | --- | --- | --- |
| S1 | Skin neoplasm* | Limits: date range - 1992-2024 | 125,000 |
| S2 | Skin cancer* | Limits: date range - 1992-2024 | 1,850,000 |
| S3 | Skin (malignant* or skin tumor* OR tumour* or carcinoma*) | Limits: date range - 1992-2024 | 18,000 |
| S4 | Cutaneous (malignant* or Skin tumour* or tumor* or carcinoma*) | Limits: date range - 1992-2024 | 18,900 |
| S5 | Melanoma* | Limits: date range - 1992-2024 | 795,000 |
| S6 | Malignan* melanoma* | Limits: date range - 1992-2024 | 329,000 |
| S7 | Basal cell carcinoma* | Limits: date range - 1992-2024 | 854,000 |
| S8 | Squamous cell carcinoma* | Limits: date range - 1992-2024 | 684,000 |
| S9 | Sebaceous cell carcinoma* | Limits: date range - 1992-2024 | 19,500 |
| S10 | Pigmented (Nevus or nevi or nevus or nevus or lesion* or mole* or skin lesion*) | Limits: date range - 1992-2024 | 12,500 |
| S11 | Suspicious (Nevus or nevi or nevus or nevus or lesion* or mole* or skin lesion*) | Limits: date range - 1992-2024 | 5,140 |
| S12 | Skin neoplasm* OR Skin cancer* OR Skin (malignant* or skin tumor* OR tumour* or carcinoma*) OR Cutaneous (malignant* or Skin tumour* or tumor* or carcinoma*) OR Melanoma* OR Malignan* melanoma* OR Basal cell carcinoma* OR Squamous cell carcinoma* OR Sebaceous cell carcinoma* OR Pigmented (Nevus or nevi or nevus or nevus or lesion* or mole* or skin lesion*) OR suspicious (Nevi or nevi or nevus or nevus or lesion* or mole* or skin lesion*) | Limits: date range - 1992-2024 | 8,080 |
| S13 | Nurse* | Limits: date range - 1992-2024 | 1,870,000 |
| S14 | "nurse led" | Limits: date range - 1992-2024 | 62,400 |
| S15 | “nurse led” OR nurse*  S14 OR S15 | Limits: date range - 1992-2024 | 2,290,000 |
| **Unable to search all term collectively** | | | |
| S16 | “skin cancer” AND nurse* | Limits: date range - 1992-2024 | 18,000 |
| S17 | skin cancer AND "nurse* led" | Limits: date range - 1992-2024 | 782 |
| S18 | “skin cancer” AND "nurse* led" | Limits: date range - 1992-2024 | 102 |

### The Cochrane library which includes both [Cochrane Central Register of Controlled Trials](https://www.cochranelibrary.com/) (CENTRAL) and Cochrane Database of Systematic Reviews (CDSR)

This table shows the search performed on 23 February 2024

| **Search ID** | **Search Terms** | **Search Options** | **Results** |
| --- | --- | --- | --- |
| S1 | MeSH descriptor: [Skin Neoplasms] Explode all trees | No limits set | 2,131 |
| S2 | Skin neoplasm* | Limits: with Cochrane Library publication date from Jan 1992 to present | 6,895 |
| S3 | Skin cancer* | Limits: with Cochrane Library publication date from Jan 1992 to present | 11,700 |
| S4 | Skin (malignan* or skin tumo?r* or carcinoma*) | Limits: with Cochrane Library publication date from Jan 1992 to present | 9,361 |
| S5 | Cutaneous (malignan* or Skin tumo?r* or carcinoma*) | Limits: with Cochrane Library publication date from Jan 1992 to present | 1,600 |
| S6 | Melanoma* | Limits: with Cochrane Library publication date from Jan 1992 to present | 6,936 |
| S7 | Malignan* (melanoma* or m* or mm*) | Limits: with Cochrane Library publication date from Jan 1992 to present | 35,615 |
| S8 | Basal cell carcinoma* | Limits: with Cochrane Library publication date from Jan 1992 to present | 1,408 |
| S9 | Squamous cell carcinoma* | Limits: with Cochrane Library publication date from Jan 1992 to present | 10,730 |
| S10 | Sebaceous cell carcinoma* | Limits: with Cochrane Library publication date from Jan 1992 to present | 14 |
| S11 | Pigmented (N?ev* or lesion*) | Limits: with Cochrane Library publication date from Jan 1992 to present | 307 |
| S12 | Suspicious (lesion* or skin lesion* or mole* or n?ev*) | Limits: with Cochrane Library publication date from Jan 1992 to present | 810 |
| S13 | S1 OR S2 OR S3 OR S4 OR S5 OR S6 OR S7 OR S8 OR S9 OR S10 OR S11 OR S12 | Limits: with Cochrane Library publication date from Jan 1992 to present | 60,247 |
| S14 | Nurs* | Limits: with Cochrane Library publication date from Jan 1992 to present | 76,068 |
| S15 | MeSH descriptor: [Nursing] explode all trees | No Limits set | 4,320 |
| S16 | S14 OR S15 | Limits: with Cochrane Library publication date from Jan 1992 to present | 76,391 |
| S17 | S13 AND S16 | Limits: with Cochrane Library publication date from Jan 1992 to present | 2,067 |
| S18 | Nurse led | Limits: with Cochrane Library publication date from Jan 1992 to present | 4,109 |
| S19 | S13 AND S16 AND S18 | Limits: with Cochrane Library publication date from Jan 1992 to present | 232 |

### Search history from ClinicalTrial.gov

This table shows the search performed on 23 February 2024

| **Search ID** | **Search Terms** | **Search Options** | **Results** |
| --- | --- | --- | --- |
| S1 | Skin cancer* | Search box entry: condition or disease | 13,203 |
| S2 | Skin neoplasm* | Search box entry: condition or disease | 13,203 |
| S3 | Melanoma* | Search box entry: condition or disease | 752 |
| S4 | Malignant melanoma | Search box entry: condition or disease | 3,306 |
| S5 | Squamous cell carcinoma* | Search box entry: condition or disease | 4,180 |
| S6 | Basal cell carcinoma* | Search box entry: condition or disease | 411 |
| S7 | Sebaceous cell carcinoma* | Search box entry: condition or disease | 7 |
| S8 | Skin (malignancy or tumours or tumors or carcinoma) | Search box entry: condition or disease | 598 |
| S9 | Cutaneous (malignancy or skin tumours or tumors or carcinoma) | Search box entry: condition or disease | 54 |
| S10 | Pigmented (nevi or naevi or nevus or naevus or lesion) | Search box entry: condition or disease | 0 |
| S11 | Suspicious (lesion or skin lesion or cutaneous lesion or mo95e or nevi or naevi or nevus or naevus) | Search box entry: condition or disease | 0 |
| S12 | Nurse | Search box entry: intervention | 5,913 |
| S13 | Skin cancer* OR Skin neoplasm* OR Melanoma* OR Malignant melanoma OR Squamous cell carcinoma* OR Basal cell carcinoma* OR Sebaceous cell carcinoma* OR Skin (malignancy or tumours or tumors or carcinoma) OR Cutaneous (malignancy or skin tumours or tumors or carcinoma) OR Pigmented (nevi or naevi or nevus or naevus or lesion) OR Suspicious (lesion or skin lesion or cutaneous lesion or mole or nevi or naevi or nevus or naevus) | Search box entry: condition or disease | 19,340 |
| S14 | Skin cancer* OR Skin neoplasm* OR Melanoma* OR Malignant melanoma OR Squamous cell carcinoma* OR Basal cell carcinoma* OR Sebaceous cell carcinoma* OR Skin (malignancy or tumours or tumors or carcinoma) OR Cutaneous (malignancy or skin tumours or tumors or carcinoma) OR Pigmented (nevi or naevi or nevus or naevus or lesion) OR Suspicious (lesion or skin lesion or cutaneous lesion or mole or nevi or naevi or nevus or naevus) AND nurse | Search box entry: condition or disease: Skin cancer* OR Skin neoplasm* OR Melanoma* OR Malignant melanoma OR Squamous cell carcinoma* OR Basal cell carcinoma* OR Sebaceous cell carcinoma* OR Skin (malignancy or tumours or tumors or carcinoma) OR Cutaneous (malignancy or skin tumours or tumors or carcinoma) OR Pigmented (nevi or naevi or nevus or naevus or lesion) OR Suspicious (lesion or skin lesion or cutaneous lesion or mole or nevi or naevi or nevus or naevus)  Intervention search box: nurse | 119 |

### Search history from World Health Organization International Clinical Trials Registry Platform

This table shows the search performed on 23 February 2024

| **Search ID** | **Search Terms** | **Results** |
| --- | --- | --- |
| S1 | skin cancer* | 637 |
| S2 | skin neoplasm* | 293 |
| S3 | melanoma* | 4,852 |
| S4 | malignan* melanoma* | 1,312 |
| S5 | squamous cell carcinoma* | 3,972 |
| S6 | sebaceous cell carcinoma* | 1 |
| S7 | basal cell carcinoma* | 460 |
| S8 | skin (malignan* or tumour* or tumor* or carcinoma*) | 9,391 |
| S9 | cutaneous malignan* skin (tumour* or tumor* or carcinoma*) | 1,642 |
| S10 | pigmented (nevi or naevi or nevus or naevus or lesion*) | 109 |
| S11 | suspicious (lesion* or skin lesion*) | 39 |
| S12 | cutaneous (lesion* or mole* or nevi or naevi or nevus or naevus) | 1,642 |
| S13 | skin cancer* OR skin neoplasm* OR melanoma* OR Malignan* melanoma* OR squamous cell carcinoma* OR sebaceous cell carcinoma* OR basal cell carcinoma* OR Skin (malignan* or tumour* or tumor* or carcinoma*) OR Cutaneous malignan* OR skin (tumour* or tumor* or carcinoma*) OR Pigmented (nevi or naevi or nevus or naevus or lesion*) OR Suspicious (lesion* or skin lesion*) OR cutaneous (lesion* or mole* or nevi or naevi or nevus or naevus) | 9,256 |
| S14 | nurs* | 7,872 |
| S15 | nurs* led | 469 |
| S16 | nurs* OR nurs* led | 7,872 |
| S17 | skin cancer OR skin neoplasm OR melanoma OR squamous cell carcinoma OR sebaceous cell carcinoma OR basal cell carcinoma OR Skin (malignancy or tumour* or tumor* or carcinoma) OR Cutaneous malignan* OR skin (tumour* or tumor* or carcinoma) OR Pigmented (nevi or naevi or nevus or naevus or lesion*) OR Suspicious (lesion* or skin lesion*) OR cutaneous (lesion* or mole* or nevi or naevi or nevus or naevus) AND nurs* OR nurs* led | 17 |

### Getting it right first time (GIRFT) website

This table shows the search performed on 23 February 2024

| **Search Terms** | **Results** |
| --- | --- |
| Dermatology, dermatology workstream  Dermatology  GIRFT Programme National Speciality report  In August 2021, GIRFT published its national speciality report for Dermatology  Getting It Right First Time GIRFT, 2021. *National specialty report for dermatology*. Available from: <https://gettingitrightfirsttime.co.uk/wp-content/uploads/2021/09/DermatologyReport-Sept21o.pdf> [Accessed 20 December 2023]. | 1 |
