# Supplementary material for: Nurse‐Led Models of Service Delivery for Skin Cancer Detection: A Systematic Review
Source: J Adv Nurs. 2025 Apr 1;81(12):8432–56. doi: 10.1111/jan.16854 (PMC12623683; doi:10.1111/jan.16854)
Supplement: Supplementary file 3 — Appendix S3. [file JAN-81-8432-s001.docx]

**Supplementary document 3**

**Joanna Briggs Institute Critical appraisal tools checklists**

**Table A:** JBI Critical Appraisal Checklist for Cohort Studies

| **Author;**  **Year; Country** | **1**  Were the two groups similar and recruited from the same population? | **2**  Were the exposures measured similarly to assign people to both exposed and unexposed groups? | **3**  Was the exposure measured in a valid and reliable way? | **4**  Were confounding factors identified? | **5**  Were strategies to deal with confounding factors stated? | **6**  Were the groups/participants free of the outcome at the start of the study (or at the moment of exposure)? | **7**  Were the outcomes measured in a valid and reliable way? | **8**  Was the follow up time reported and sufficient to be long enough for outcomes to occur? | **9**  Was follow up complete, and if not, were the reasons to loss to follow up described and explored? | **10**  Were strategies to address incomplete follow up utilized? | **11**  Was appropriate statistical analysis used? | **Overall Appraisal and Outcome** |
| --- | --- | --- | --- | --- | --- | --- | --- | --- | --- | --- | --- | --- |
| Jones, Jameson, & Oakley, 2021; New Zealand. Remote Skin Cancer Diagnosis: Adding Images to Electronic Referrals Is More Efficient Than Wait-Listing for a Nurse-Led Imaging Clinic | YES | UNCLEAR | UNCLEAR | YES | NO | YES | YES | YES | YES | UNCLEAR | YES | MODERATE  QUALITY INCLUDE |
| Lim, Oakley, & Rademaker, 2012; New Zealand. Better, sooner, more convenient: A successful teledermoscopy service | NO | UNCLEAR | YES | UNLCEAR | UNCLEAR | YES | YES | UNCLEAR | UNCLEAR | UNCLEAR | UNCLEAR | LOW QUALITY  INCLUDE |

**Table B:** JBI Critical Appraisal Checklist for Quasi-Experimental studies

| **Author;**  **Year; Country** | **1**  **Is it clear in the study what is the ‘cause’ and what is the ‘effect’ (i.e. there is no confusion about which variable comes first)?** | **2**  **Were the participants included in any comparisons similar?** | **3**  **Were the participants included in any comparisons receiving similar treatment/care, other than the exposure or intervention of interest?** | **4**  **Was there a control group?** | **5**  **Were there multiple measurements of the outcome both pre and post the intervention/exposure?** | **6**  **Was follow up complete and if not, were differences between groups in terms of their follow up adequately described and analyzed?** | **7**  **Were the outcomes of participants included in any comparisons measured in the same way?** | **8**  **Were outcomes measured in a reliable way?** | **9**  **Was appropriate statistical analysis used?** | **Overall Appraisal and Outcome** |
| --- | --- | --- | --- | --- | --- | --- | --- | --- | --- | --- |
| Clayton, Tait, Whitehurst, & Yates, 2006; England. Photodynamic therapy for superficial basal cell carcinoma and Bowen's disease | YES | YES | UNCLEAR | YES | NO | YES | YES | UNCLEAR | UNCLEAR | MODERATE  QUALITY INCLUDE |

**Table C:** JBI Critical Appraisal Checklist for Case-Control studies

| **Author;**  **Year; Country** | **1**  **Were the groups comparable other than the presence of disease in cases or the absence of disease in controls?** | **2**  **Were cases and controls matched appropriately?** | **3**  **Were the same criteria used for identification of cases and controls?** | **4**  **Was exposure measured in a standard, valid and reliable way?** | **5**  **Was exposure measured in the same way for cases and controls?** | **6**  **Were confounding factors identified?** | **7**  **Were strategies to deal with confounding factors stated?** | **8**  **Were outcomes assessed in a standard, valid and reliable way for cases and controls?** | **9**  **Was the exposure period of interest long enough to be meaningful?** | **10**  **Was appropriate statistical analysis used?** | **Overall Appraisal and Outcome** |
| --- | --- | --- | --- | --- | --- | --- | --- | --- | --- | --- | --- |
| Oliveria, Dusza, Phelan, Ostroff, Berwick, & Halpern, 2004; United States of America. Patient Adherence to Skin Self-Examination Effect of Nurse Intervention with Photographs | YES | YES | YES | UNCLEAR | YES | UNCLEAR | UNCLEAR | YES | NO | YES | MODERATE  QUALITY INCLUDE |

**Table D:** JBI assessment for Case-Series studies

| **Author;**  **Year; Country** | **1**  **Were there clear criteria for inclusion in the case series?** | **2**  **Was the condition measured in a standard, reliable way for all participants included in the case series?** | **3**  **Were valid methods used for identification of the condition for all participants included in the case series?** | **4**  **Did the case series have consecutive inclusion of participants?** | **5**  **Did the case series have complete inclusion of participants?** | **6**  **Was there clear reporting of the demographics of the participants in the study?** | **7**  **Was there clear reporting of clinical information of the participants?** | **8**  **Were the outcomes or follow up results of cases clearly reported?** | **9**  **Was there clear reporting of the presenting site(s)/clinic(s) demographic information?** | **10**  **Was statistical analysis appropriate?** | **Overall Appraisal and Outcome** |
| --- | --- | --- | --- | --- | --- | --- | --- | --- | --- | --- | --- |
| Jones and Mullen, 2014; England. A service evaluation of a nurse consultant-led basal cell carcinoma clinic. | YES | YES | YES | YES | YES | NO | YES | YES | UNCLEAR | UNCLEAR | HIGH QUALITY  INCLUDE |
| Mohite, et al., 2016; England. Accuracy of clinical diagnosis of benign eyelid lesions: Is a dedicated nurse-led service safe and effective? | YES | YES | YES | YES | YES | YES | YES | YES | YES | YES | HIGH QUALITY  INCLUDE |
