# Supplementary material for: Nurse‐Led Models of Service Delivery for Skin Cancer Detection: A Systematic Review
Source: J Adv Nurs. 2025 Apr 1;81(12):8432–56. doi: 10.1111/jan.16854 (PMC12623683; doi:10.1111/jan.16854)
Supplement: Supplementary file 4 — Appendix S4. [file JAN-81-8432-s002.docx]

**Supplementary document 4**

**Articles Meeting Inclusion Criteria**

Clayton, T. H., Tait, J., Whitehurst, C., and Yates, V. M. 2006. Photodynamic therapy for superficial basal cell carcinoma and Bowen's disease. *European journal of dermatology*, 16 (1), 39–41.

Jones L, Jameson M, Oakley A. Remote Skin Cancer Diagnosis: Adding Images to Electronic Referrals Is More Efficient Than Wait-Listing for a Nurse-Led Imaging Clinic. *Cancers,* 13 (22), 5828.

Jones, C. and Mullen, L. 2014. A service evaluation of a new nurse consultant-led basal cell carcinoma clinic. *Dermatological Nursing*, 13 (3), 39–44.

Lim, D., Oakley, A. M. M., and Rademaker, M. 2012. Better, sooner, more convenient: a successful teledermoscopy service. *Australasian Journal of Dermatology*, 53 (1), 22–25.

Mohite, A. A., Johnson, A., Rathore, D. S., Bhandari, K., Crossman, R., Mehta, P., and Ahluwalia, H. S. (2016). Accuracy of clinical diagnosis of benign eyelid lesions: Is a dedicated nurse-led service safe and effective?. *Orbit*, 35 (4), 193–198.

Oliveria, S. A., Dusza, S. W., Phelan, D. L., Ostroff, J. S., Berwick, M., and Halpern, A. C., 2004. Patient adherence to skin self-examination. effect of nurse intervention with photographs. *American journal of preventive medicine*, 26 (2), 152–155.
